# Targeting the glycine-rich domain of TDP-43 with antibodies prevents its aggregation in vitro and reduces neurofilament levels in vivo

**DOI:** 10.1186/s40478-023-01592-z

**Published:** 2023-07-11

**Authors:** Henrick Riemenschneider, Francesca Simonetti, Udit Sheth, Eszter Katona, Stefan Roth, Saskia Hutten, Daniel Farny, Meike Michaelsen, Brigitte Nuscher, Michael K. Schmidt, Andrew Flatley, Aloys Schepers, Lara A. Gruijs da Silva, Qihui Zhou, Thomas Klopstock, Arthur Liesz, Thomas Arzberger, Jochen Herms, Regina Feederle, Tania F. Gendron, Dorothee Dormann, Dieter Edbauer

**Affiliations:** 1 German Center for Neurodegenerative Diseases (DZNE), Munich, Feodor-Lynen-Str. 17, 81377 Munich, Germany; 2 Munich Cluster of Systems Neurology (SyNergy), Feodor-Lynen-Str. 17, 81377 Munich, Germany; 3 Ludwig-Maximilians-Universität (LMU) Munich, Graduate School of Systemic Neurosciences (GSN), 81377 Munich, Germany; 4 Institute for Stroke and Dementia Research (ISD), University Hospital, LMU Munich, Feodor-Lynen-Str. 17, 81377 Munich, Germany; 5 Institute of Molecular Physiology, Faculty of Biology, Johannes Gutenberg-Universität (JGU), Hanns-Dieter-Hüsch-Weg 17, 55128 Mainz, Germany; 6 Department of Neuroscience, Mayo Clinic, 4500 San Pablo Road, Jacksonville, FL 32224 USA; 7 Mayo Clinic Graduate School of Biomedical Sciences, Mayo Clinic, Jacksonville, FL 32224 USA; 8 Chair of Metabolic Biochemistry, Biomedical Center (BMC), Faculty of Medicine, Ludwig-Maximilians-Universität (LMU) Munich, Feodor-Lynen-Str. 17, 81377 Munich, Germany; 9 Center for Neuropathology and Prion Research, University Hospital, LMU Munich, Feodor-Lynen-Str. 23, 81377 Munich, Germany; 10 Monoclonal Antibody Core Facility, Helmholtz Zentrum München, German Research Center for Environmental Health (GmbH), Ingolstädter Landstr. 1, 85764 Neuherberg, Germany; 11 Friedrich Baur Institute at the Department of Neurology, University Hospital, LMU Munich, Ziemssenstr. 1a, 80336 Munich, Germany; 12 Department of Psychiatry and Psychotherapy, University Hospital, LMU Munich, Nußbaumstr. 7, 80336 Munich, Germany; 13 Institute of Molecular Biology (IMB), Ackermannweg 4, 55128 Mainz, Germany

**Keywords:** Neurodegeneration, Amyotrophic lateral sclerosis, Frontotemporal dementia, TDP-43, Immunotherapy, Phase separation, Aggregation

## Abstract

**Abstract:**

Cytoplasmic aggregation and concomitant nuclear clearance of the RNA-binding protein TDP-43 are found in ~ 90% of cases of amyotrophic lateral sclerosis and ~ 45% of patients living with frontotemporal lobar degeneration, but no disease-modifying therapy is available. Antibody therapy targeting other aggregating proteins associated with neurodegenerative disorders has shown beneficial effects in animal models and clinical trials. The most effective epitopes for safe antibody therapy targeting TDP-43 are unknown. Here, we identified safe and effective epitopes in TDP-43 for active and potential future passive immunotherapy. We prescreened 15 peptide antigens covering all regions of TDP-43 to identify the most immunogenic epitopes and to raise novel monoclonal antibodies in wild-type mice. Most peptides induced a considerable antibody response and no antigen triggered obvious side effects. Thus, we immunized mice with rapidly progressing TDP-43 proteinopathy (“rNLS8” model) with the nine most immunogenic peptides in five pools prior to TDP-43ΔNLS transgene induction. Strikingly, combined administration of two N-terminal peptides induced genetic background-specific sudden lethality in several mice and was therefore discontinued. Despite a strong antibody response, no TDP-43 peptide prevented the rapid body weight loss or reduced phospho-TDP-43 levels as well as the profound astrogliosis and microgliosis in rNLS8 mice. However, immunization with a C-terminal peptide containing the disease-associated phospho-serines 409/410 significantly lowered serum neurofilament light chain levels, indicative of reduced neuroaxonal damage. Transcriptomic profiling showed a pronounced neuroinflammatory signature (IL-1β, TNF-α, NfκB) in rNLS8 mice and suggested modest benefits of immunization targeting the glycine-rich region. Several novel monoclonal antibodies targeting the glycine-rich domain potently reduced phase separation and aggregation of TDP-43 in vitro and prevented cellular uptake of preformed aggregates. Our unbiased screen suggests that targeting the RRM2 domain and the C-terminal region of TDP-43 by active or passive immunization may be beneficial in TDP-43 proteinopathies by inhibiting cardinal processes of disease progression.

**Graphical Abstract:**

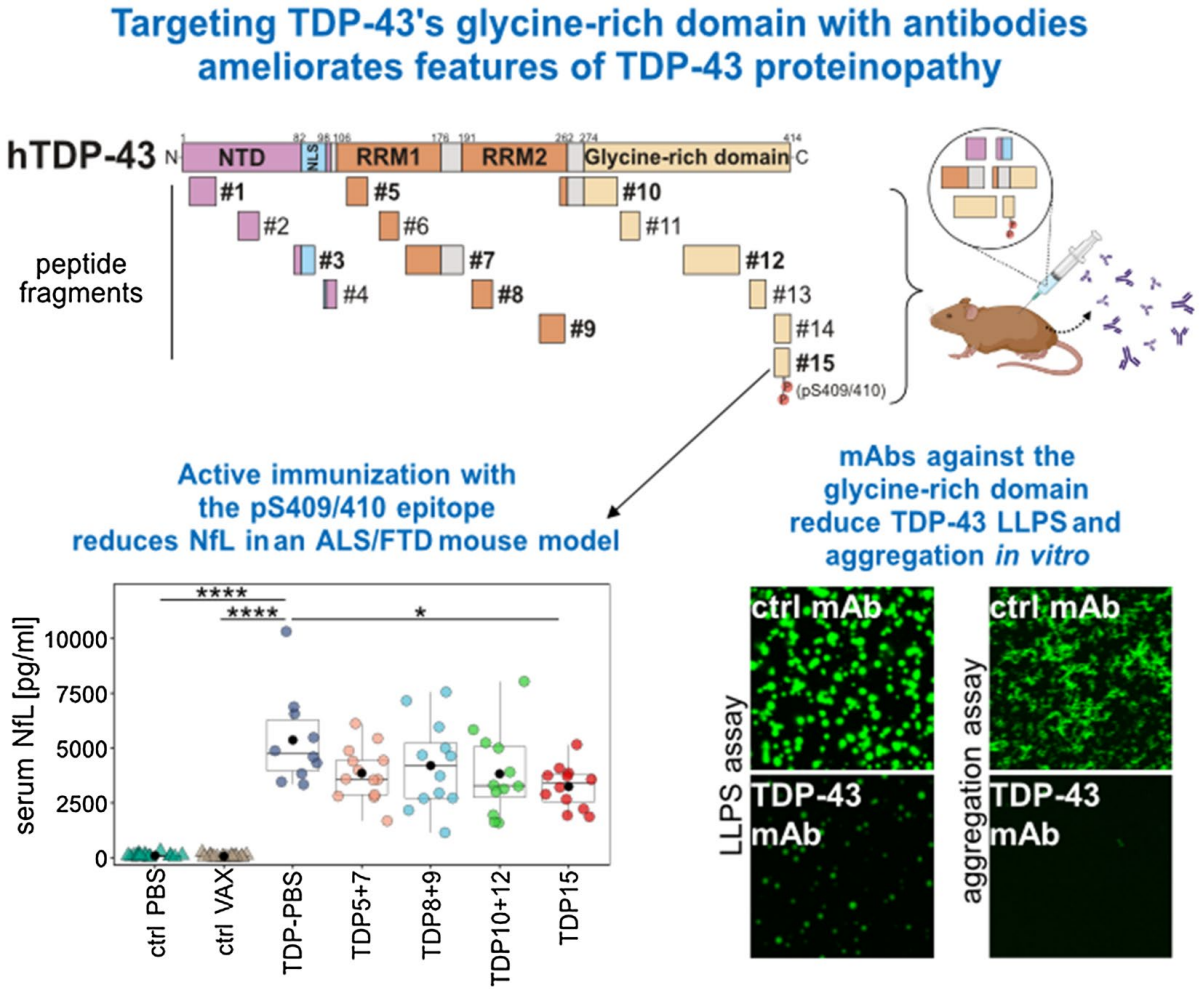

**Supplementary Information:**

The online version contains supplementary material available at 10.1186/s40478-023-01592-z.

## Introduction

Amyotrophic lateral sclerosis (ALS) and frontotemporal lobar degeneration with TDP-43 inclusions (FTLD-TDP) are related, incurable neurodegenerative disorders with overlapping genetics and neuropathology. Most cases show neuronal and glial cytoplasmic inclusions of the multifunctional RNA-binding protein TDP-43 [30]. Normally, TDP-43 resides mostly in the nucleus where it regulates gene expression at multiple levels with a dominant role in mRNA splicing [46, 55]. TDP-43 inclusions show disease-specific posttranslational modifications, such as proteolytic processing into aggregation-prone 25–35 kDa C-terminal fragments, phosphorylation and ubiquitination [18, 37, 38]. In contrast to nuclear TDP-43, cytoplasmic inclusions are hyperphosphorylated at serines 409/410 (pS409/410), but the pathophysiological role of C-terminal TDP-43 phosphorylation is still debated [16, 32]. The distribution of TDP-43 pathology strongly correlates with neuron loss in affected central nervous system (CNS) regions of ALS and FTLD-TDP cases, underpinning its key role in the pathophysiology of these diseases [29]. Most aggregate-bearing cells show striking clearance of nuclear TDP-43, suggesting that gain and loss of TDP-43 function pathomechanisms act in concert [27]. In mice, both knockout and overexpression of wild-type TDP-43 are lethal, raising concerns about the safety of TDP-43 targeted therapy [12, 59].

Over the past years, active and passive antibody approaches targeting different extracellular (Aβ) and intracellular (Tau, α-synuclein, poly-GA) aggregating proteins have emerged as promising options for the treatment of neurodegenerative diseases [34]. Despite limited permeability of the blood brain barrier for antibodies, Aβ-specific antibodies reduce amyloid load, and two such antibodies are now FDA-approved for the treatment of Alzheimer’s disease, though their clinical utility is still debated. Ongoing antibody therapy approaches for intracellular aggregating proteins have been suggested to prevent their unconventional cell-to-cell transmission, thus inhibiting pathogenic spreading and promoting clearance by microglia or preventing toxic neuroinflammation [21, 63]. In addition, low level intracellular antibody uptake may accelerate proteasomal clearance of aggregates through recruitment of the intracellular Fc-receptor TRIM21 [33, 35].

For intracellular Tau aggregates, both passive antibody delivery and active vaccination are currently being tested in clinical trials [40]. We have used active vaccination to target the abundant poly-GA inclusions found in ALS and FTLD cases caused by *C9orf72* repeat expansions [63]. Immunization of presymptomatic mice expressing poly-GA with ovalbumin (OVA)-coupled (GA)_10_ induced a strong anti-GA response, reduced poly-GA aggregation, prevented microgliosis, and improved motor deficits [63]. For TDP-43, the Julien lab has generated a monoclonal antibody directed against its RNA recognition motif 1 (RRM1) that reduced cytoplasmic TDP-43 levels as well as TDP-43/p65-mediated NFκB activation in vitro. Intrathecal injection of this antibody ameliorated cytoplasmic TDP-43 mislocalization in the lumbar spinal cord, which was accompanied by microglial activation [44]. Expressing the same antibody in the brain as a single-chain variable fragment (scFv) using AAV also reduced TDP-43 proteinopathy, accompanying neuroinflammation, and behavioral abnormalities in BAC-transgenic TDP-43 mice [45]. Two other scFv antibodies directed against an epitope in the RRM2 domain were genetically engineered to promote TDP-43 aggregate clearance in cell lines and validated in mice using combined in utero electroporation of TDP-43 and scFv [53]. However, active vaccination targeting TDP-43 has not been reported, although this approach would be more convenient and cost-efficient in patients.

Given the importance of TDP-43-associated neurodegeneration in ALS and FTLD-TDP as well as the recent advances in immunotherapies for neurodegenerative disorders, we tested whether active vaccination would ameliorate TDP-43 pathophysiology in vivo. The choice of epitope for active and passive immunotherapy is challenging, because the domains driving gain-of-function pathomechanisms are unclear and it is still debated whether TDP-43 inclusions are liquid-like, gel-like or amyloid-like [5, 13, 26, 49, 61]. To identify the best target epitopes in vivo, we screened peptide antigens covering all regions of TDP-43 for active immunization, and investigated their effects on TDP-43 phosphorylated at serines 409/410 (pTDP-43), transcriptional alterations, neuroinflammation, and neuroaxonal damage in an aggressive mouse model of TDP-43 proteinopathy. In addition, we raised and characterized a panel of monoclonal antibodies from the immunized mice. Altogether, we found active vaccination targeting the C-terminal low-complexity domain, in particular the C-terminal pS409/410 epitope, to have moderate beneficial effects in vivo, and monoclonal antibodies targeting the C-terminal low-complexity domain to potently suppress phase separation, aggregation and aggregate uptake of TDP-43 in vitro, commending this region for future antibody-based therapeutic efforts.

## Materials and methods

### Mice and immunization regimen for active vaccination study

All animal procedures were performed according to institutional guidelines approved by the governmental ethics committee of Upper Bavaria (licenses TV 55.2-2532.Vet_02-17-106 and TV 55.2-2532.Vet_03-17-68). Mice were housed in standard IVC green line cages (Tecniplast, Italy) on a 12-h light/dark cycle in a pathogen-free facility and ad libitum access to food and water. The monogenic lines B6;C3-Tg(NEFH-tTA)8Vle/J (NEFH-tTA line 8, stock #025397) and B6;C3-Tg(tetO-TARDBP*)4Vle/J (tetO-hTDP-43ΔNLS line 4, stock #014650) were obtained from the Jackson Laboratory (Bar Harbour, USA) and intercrossed to generate bigenic regulatable NLS8 (rNLS8) animals with a mixed C57BL/6J × C3H/HeJ background. Breeders as well as offspring mice were kept on a doxycycline diet (200 mg/kg, Ssniff, Germany) during hTDP-43∆NLS suppression (until aged to 25.5 weeks). To induce hTDP-43∆NLS expression, animals were switched to standard chow lacking doxycycline (Ssniff, Germany). For genotyping, DNA was extracted from ear biopsies by the previously described HotSHOT method [54] and transgenes were detected by PCR using the primers recommended by Jackson Laboratory: NEFH-tTA forward 5′-CTCGCGCACCTGCTGAAT-3′, NEFH-tTA reverse 5′-CAGTACAGGGTAGGCTGCTC-3′, tetO-TARDBP* forward 5′-TTGCGTGACTCTTTAGTATTGGTTTGATGA-3′, tetO-TARDBP* reverse 5′-CTCATCCATTGCTGCTGCG-3′. Littermates were grouped into indicated immunization cohorts in a randomized and gender-balanced fashion.

**Fig. 1:**
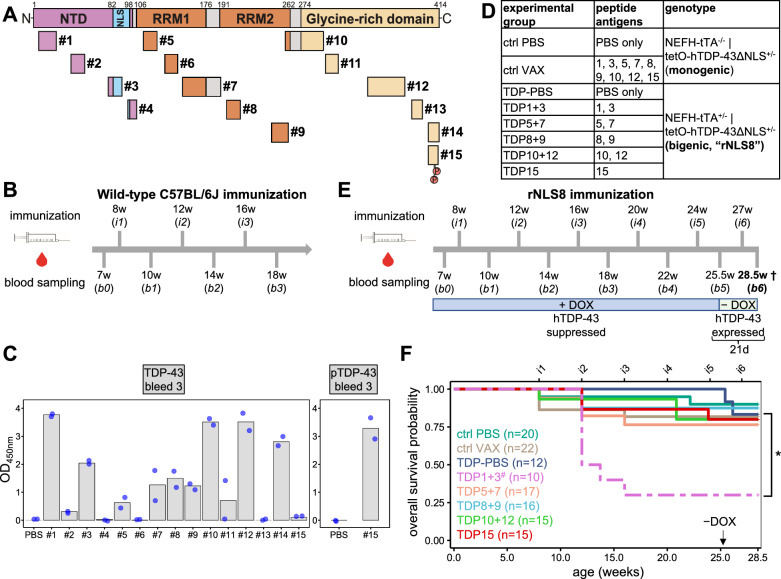
Epitope scan of TDP-43 reveals differential immunogenicity and safety in mice. Active immunization with 15 different TDP-43 peptides in wild-type C57BL/6J to select the most immunogenic ones for therapeutic vaccination in rNLS8 mice. **A** Localization and relative size of peptides used for active immunization targeting human TDP-43. Peptide 15 contains pS409/410. NTD: N-terminal domain, NLS: Nuclear localization signal, RRM1/2: RNA recognition motif 1/2. **B** Immunization regimen for wild-type C57BL/6J mice (age in weeks). Two mice per peptide antigen were immunized monthly (i1-i3) with serum collected before and in between immunizations (b0-b3). **C** Antibody response towards human recombinant TDP-43 (left panel) or pTDP-43 peptide (pS409/410, right panel) was determined by ELISA after three immunizations. Background-corrected optical density at 450 nm (OD450) was measured in duplicates for each animal (dots) with bars representing mean values. All sera were diluted 1:500. Preimmune serum is analyzed in Fig. S1B. **D** Experimental groups for therapeutic vaccination. rNLS8 animals expressing hTDP-43ΔNLS were immunized with either pooled antigens or PBS control. Monogenic littermates received either PBS (ctrl PBS) or a mixture of all peptides used for rNLS8 immunization (ctrl VAX). Peptides 1 and 3 were excluded from the control group after discovering side effects in the TDP1 + 3 group (Fig. 1F). **E** Timeline of immunization and blood sampling in rNLS8 and control mice. After the fifth blood sampling (b5), hTDP-43∆NLS expression was induced by removing doxycycline (DOX) from the chow. All mice were sacrificed 21 days after transgene induction (28.5 weeks of age). **F** Kaplan–Meier plot showing survival probability of mice during study (humane endpoint). Number of animals at the beginning of the immunization regimen is indicated. Log-rank test and pairwise comparisons with Benjamini–Hochberg correction revealed a statistically significant higher mortality in TDP1 + 3 immunized compared to TDP-PBS control mice (*, *p* = 0.039). Note that immunization of surviving TDP1 + 3 mice was discontinued after 1, 2 or 3 immunizations (^#^, dashed line)

Immunization followed our previous protocol for poly-GA vaccination [63]. For the initial immunization of wild-type C57BL/6J mice, a total of 40 µg ovalbumin peptide conjugates (Peptide Specialty Laboratories GmbH, Germany) were mixed with 5 nmol CpG ODN 1668 oligonucleotide (IAX-200–001, Innaxon, UK) in 200 µl PBS and 250 µl Incomplete Freund’s adjuvant (IFA, Sigma, USA) and injected half-half intraperitoneally (i.p.) and subcutaneously (s.c.) into 8-week-old animals. For the monthly booster immunizations, a total of 40 µg antigen was mixed with 5 nmol CpG ODN 1668 in 500 µl PBS and injected half-half i.p. and s.c. as depicted in Fig. 1B. rNLS8 immunization followed the same regimen (Fig. 1E), but using 250 µl Alhydrogel® adjuvant 2% (Invivogen, USA). The control groups (wild-type C57BL/6J: PBS; rNLS8: ctrl PBS and TDP-PBS) received PBS instead of the peptide conjugates. Immunizations in CD-1 (#022), C3H/HeOuJ (#626) and C57BL/6J (#632, all three lines obtained from Charles River Laboratories, Sulzfeld, Germany) were carried out as described for rNLS8 mice (Additional file 1: Fig. S2A).

### Serum collection and tissue harvesting

Monthly blood collection was carried out by puncture of the vena facialis using a Goldenrod animal lancet (BioMedical Instruments Trading, Germany). Blood clotted during 15 min incubation at room temperature (RT) and serum was obtained as the supernatant after centrifugation (13,000× *g* for 10 min at 4 °C). Three weeks after transgene induction, an overdosed combination of ketamine (450 mg/kg, Ketabel, belapharm, Germany) with xylazine (30 mg/kg, Xylazin, Serumwerk, Germany) was intraperitoneally injected into rNLS8 and control mice to ensure painless deep anesthesia without recovery, thereby allowing for transcardial perfusion using ice-cold phosphate-buffered saline (DBPS, Gibco, USA). Collected tissue was either snap-frozen in liquid nitrogen and stored at −80 °C for subsequent biochemical analysis or formalin-fixed (ROTI®Histofix 4%, Roth, Germany) for 24 h for immunohistochemistry/immunofluorescence.

### TDP-43 antibody ELISA

TDP-43 antibody titers in mouse sera and the binding affinities of purified monoclonal antibodies to TDP-43 were determined by ELISA. To this end, Nunc MaxiSorp™ flat bottom 96-well plates were incubated with recombinant human TDP-43-MBP-His_6_ or a pTDP-43 (pS409/410) peptide (biotinylated version of TDP peptide #15) in PBS overnight at 4 °C. The next day, plates were treated with blocking solution (1% BSA, 0.05% Tween 20 in PBS) for 1 h at RT. Subsequently, serum samples were prediluted in blocking solution as depicted in the figure legends and added for 1 h at RT. Following three washes with 0.05% Tween 20 in PBS, plates were incubated with anti-mouse secondary antibody (Promega, USA) for 1 h at RT. After three more washes, 3,3′,5,5′-Tetramethylbenzidine (TMB, Sigma, USA) was used as chromogenic substrate and its reaction was stopped using 2 M H_2_SO_4_. The optical density at 450 nm (OD450) was measured using a Biotek Cytation 3 plate reader. Background signals were determined by using MBP-His_6_ (for TDP-43-MBP-His_6_) or PBS (for pTDP-43) as coating agents. Out-of-range signals were manually set to the detection maximum of 4. OD signals of two technical replicates per sample were background-corrected, averaged, and plotted.

### Mesoscale discovery immunoassays for pTDP-43 and inflammatory markers

Levels of pTDP-43 and inflammatory markers were measured using the Mesoscale Discovery platform (USA). To generate brain lysates, approximately 50 mg of neocortex tissue from mice was homogenized in cold RIPA buffer (50 mM Tris–HCl pH 8.0, 150 mM NaCl, 5 mM EDTA, 0.5% sodium deoxycholate, 1% Nonidet P-40, 0.1% sodium dodecyl sulfate, and protease and phosphatase inhibitors) and sonicated on ice. Homogenates were centrifuged at 100,000 × g for 30 min at 4 °C and the supernatant was collected as RIPA-soluble fraction. The RIPA-insoluble pellet was extracted using 7 M urea buffer (7 M urea, 2 M thiourea, 4% CHAPS, 30 mM Tris–HCl pH 8.5), agitated vigorously at RT for 30 min, and centrifuged at 100,000 × g for 30 min at 22 °C. The protein concentrations of the RIPA-soluble and urea-soluble fractions were determined by bicinchoninic acid (BCA) and Bradford assay, respectively.

Levels of pTDP-43 were measured on a homebrew immunoassay performed as previously described [47]. The capture antibody was a mouse monoclonal antibody that detects TDP-43 phosphorylated at serines 409/410 (1:500, TIP-PTD-M01, Cosmo Bio, USA), and the detection antibody was a sulfo-tagged rabbit polyclonal C-terminal TDP-43 antibody (2 μg/ml, 12892-1-AP, Proteintech). Samples were diluted in TBS (Tris-buffered saline) to 35 μg protein per well and tested in duplicate wells. Both RIPA-soluble and RIPA-insoluble fractions were quantified. Response values corresponding to the intensity of emitted light upon electrochemical stimulation of the assay plate using the MSD QUICKPLEX SQ120 were acquired.

Pro-inflammatory markers and cytokine levels were quantified using V-PLEX Plus Mouse Cytokine 19-Plex Kit (K15255G-2, Mesoscale Discovery) according to the manufacturer’s protocol using the RIPA-soluble fraction. 650 µg and 250 µg total protein were loaded per well on the pro-inflammatory and cytokine multiplex plate, respectively. Markers for which all measurements were below the detection range determined by the standard curve were neglected. For all other robustly working assays, concentration values below detection range were set to zero. Measurements with a CV of > 20% were excluded.

### HEK293 CRISPR interference (CRISPRi) *TARDBP* knockdown cells

HEK293 cells were a gift from Martin Dichgans (Institute for Stroke and Dementia Research, University Hospital, LMU Munich) and CRISPRi single cell clones were generated as described before [31]. In detail, HEK293 cells were cultured in DMEM (Thermo Fisher, USA) supplemented with 10% tetracycline-free fetal bovine serum (Clontech, USA) and 1% Non-Essential Amino Acids (Thermo Fisher). Transgenic lines were generated by co-transfecting cells in a 1:1:2.5 ratio using the following constructs: hAAVS1 1L TALEN (addgene #35431), hAAVS1 1R TALEN (addgene #35432), and pAAVS1-NDi-CRISPRi (Gen1, addgene #73497). This allows integration of the Cas9 cassette under the human safe harbor locus AAVS1 and expression of the fusion protein [Krüppel-associated box (KRAB) repressor domain fused to deactivated Cas9 (dCas9) and a mCherry marker] in a doxycycline-inducible manner. To generate single-cell clones, transfected cells were seeded in serial dilutions under G418 (400 µg/ml) selection and cultured until colonies were visible. After expansion, only single cell clones that showed a uniform and efficient dCas9 induction with tight regulation were used in later experiments. Next, these clones were transfected with a gRNA expression plasmid targeting *TARDBP* (sgRNA sequence: 5′-CAGGGACACCGAAGCAGCGA-3′) and clonally selected using Hygromycin (200 µg/ml). Doxycycline-dependent *TARDBP* knockdown was evaluated by immunoblotting and the most efficient clones were used further. For testing mouse antisera in immunoblotting, cells were grown in the absence or presence of 2 µM doxycycline for 72 h, washed with PBS, and lysed on ice in RIPA (137 mM NaCl, 20 mM Tris–HCl pH 7.5, 0.1% SDS, 10% glycerol, 1% Triton X-100, 0.5% deoxycholate, 2 mM EDTA) supplemented with Benzonase Nuclease (67 U/ml), protease inhibitor cocktail, and phosphatase inhibitor cocktail (all from Sigma, USA). Lysates were centrifuged (18,000 × g for 30 min at 4 °C) and the protein concentration of the RIPA-soluble supernatant was determined using a BCA assay (Interchim, France). After adding 3 × loading buffer (200 mM Tris–HCl pH 6.8, 6% SDS, 20% glycerol, 0.1 g/ml DTT, 0.1 mg Bromophenol Blue), samples were heat denatured at 95 °C for 10 min. Equivalent protein amounts were loaded on 10% SDS–PAGE gels and transferred to Nitrocellulose membranes (GE Healthcare, USA). Membranes were blocked in 5% milk powder and probed with a commercial anti-TDP-43 antibody (1:500, 10782–2-AP, Proteintech) or pooled antisera from five-times immunized mice (bleed 5). An anti-Calnexin antibody (1:7000, ADI-SPA-860, Enzo Life Sciences) was used to detect Calnexin as the loading control protein.

### Automated immunohistochemistry (IHC) and immunofluorescence (IF) stainings, image quantification

Immunohistochemistry (chromogenic detection) and immunofluorescence (fluorescent detection) stainings of mouse brain tissue were performed on formalin-fixed, paraffin-embedded (FFPE) sections of 5 µm thickness using automated staining systems with the following primary antibodies: pTDP-43 (pS409/410) (1:50, clone 1D3, Helmholtz Center Munich), TDP-43 (1:200, 10782–2-AP, Proteintech), Iba1 (1:250, 234308, SYSY), and GFAP (1:500, 173006, SYSY).

Immunohistochemical stainings of pTDP-43 (pS409/410) were performed on a Ventana BenchMark Ultra automated stainer using pretreatment with CC1 buffer and the ultraView Universal DAB Detection Kit (all from Roche). Nuclei were counterstained using hematoxylin. All steps followed standard manufacturer’s instructions. Acquisition of bright-field images was performed on a Leica Dmi8 microscope equipped with a HC PL FLUOTAR L 20x/0.40 DRY objective and a DMC4500 camera (Leica Microsystems, Germany). All images were captured using the LAS X software (version 3.7) in tile-scan mode at a 1280 × 960 pixel resolution.

Immunofluorescence stainings for TDP-43, Iba1, and GFAP were performed on a DCS Delta.36 automated staining system using supplier reagents (DCS-Diagnostics, Germany). All steps were conducted at RT. After deparaffinization, the TR2 (low pH) buffer was used for antigen retrieval. Sections were then blocked and permeabilized using 2% fetal calf serum, 3% goat serum, and 0.2% Triton X-100 in PBS. Next, primary antibodies were added for 1.5 h, followed by an 1 h incubation step with host-specific secondary antibodies (each 1:250, goat anti-rabbit Alexa 488, goat anti-guinea pig Alexa 555, goat anti-chicken Alexa 647; all from Thermo Fisher). Afterwards, nuclei were counterstained using DAPI (1:5000 in PBS, 15 min) and sections were mounted with Vectashield Vibrance (VEC-H-1700, Biozol, Germany). Stained sections were imaged on a PANNORAMIC “MIDI II” Digital Slide Scanner (3DHistech, Hungary) equipped with a 20 × objective and the Pannoramic Slide Scanner software (version 2.0.5). To quantify Iba1 and GFAP signals automatically, a customized ImageJ/Fiji (version 1.53t) script to detect cell soma was used. In brief, images were background-subtracted (rolling ball method, radius 50 pixels) and despeckled (median filter) first. Next, Morphological Filters and Gray Scale Attribute Filtering (both from the MorphoLibJ plugin, version 1.6.0) were applied, followed by auto thresholding (Iba1: IJ_IsoData; GFAP: auto local threshold, Phansalkar method). Cell soma were then counted using the “Analyze Particles” function (size Iba1: 15–150 pixels^2^; size GFAP: 50–200 pixels^2^). Afterwards, cell counts were normalized to the hippocampal area analyzed.

Images of TDP-43 (human + mouse) stained sections were acquired on an inverted LSM710 Axio Observer.Z1 confocal laser scanning microscope equipped with a Plan-Apochromat 40x/1.40 Oil DIC M27 objective and a PMT detector. Images were taken at 2048 × 2048 pixel resolution in z-stack mode (step size 0.42 µm) using the ZEN 2011 software (black edition, Carl Zeiss, Germany). For each animal investigated, three fields of view in the frontal cortex area were acquired. Using CellPose (version 2.2) [41, 52], nuclear (based on DAPI staining) and pan-TDP-43 (based on TDP-43 staining) signals were segmented (cyto2 model, cell diameter = 100 pixels, flow threshold = 0.4). Z-series were projected into two dimensions using Image J’s sum slices method. Based on the outlines obtained from CellPose segmentation, nuclear and cytoplasmic (non-nuclear) TDP-43 intensities were measured, and their ratio was calculated. Ratios of the three fields of view were averaged and plotted as depicted in Fig. 4C.

### Patient tissue, immunofluorescence

Patient brain sections for immunofluorescence stainings were provided by the Neurobiobank Munich and Ludwig-Maximilians-University (LMU) Munich. Their usage was reviewed and approved by the ethical committee at LMU Munich. For immunofluorescence staining, 5 µm-thick formalin-fixed and paraffin-embedded sections from the Gyrus frontalis medius region were deparaffinized and rehydrated using a standard xylene/ethanol gradient. Heat-mediated antigen retrieval was performed for 30 min in citrate buffer pH 6.0 using a boiling steamer. Next, sections were treated with blocking buffer (2% fetal calf serum, 3% goat serum, 0.2% Triton X-100 in PBS) and incubated for 1 h at RT with either a commercially available primary antibody (pTDP-43 (pS409/410), 80007–1-RR, 1:500 or TDP-43, 10782–2-AP, 1:350, both from Proteintech), pooled blood sera from five-times immunized mice (bleed 5) or the purified TDP-43 monoclonal antibodies, each diluted in blocking solution (0.2% fetal calf serum, 0.2% bovine serum albumin, 0.02% fish gelatine in PBS). Subsequently, tissues were incubated with goat anti-mouse Alexa 488- and goat anti-rabbit Alexa 555-coupled secondary antibodies (each 1:400, both from Thermo Fisher) for 1 h at RT. After each incubation step, three washes with 0.05% Tween 20 in PBS were performed. DAPI was used to counterstain nuclei (1:5000 in PBS, 10 min, RT) and sections were briefly incubated with Sudan black solution to reduce autofluorescence. Lastly, tissue sections were mounted using Vectashield Vibrance (VEC-H-1700, Biozol, Germany) and imaged with an inverted LSM710 Axio Observer.Z1 confocal laser scanning microscope equipped with a Plan-Apochromat 63x/1.40 Oil DIC M27 objective and a PMT detector. Images were acquired at 2048 × 2048 pixel resolution with unidirectional scanning using two-fold frame averaging and the ZEN 2011 software (black edition, Carl Zeiss, Germany).

### Neurofilament light chain (NfL) measurement

Neurofilament light chain (NfL) levels in blood sera were quantified using the Simoa NF-light Advantage Kit (103186, Quanterix, USA). Sera were diluted 1:40 (v/v) in sample dilution buffer and measured on a Simoa HD-1 Analyzer (Quanterix) following manufacturer’s instructions. NfL serum concentration was interpolated from standard curves and mean values from duplicate measurements were plotted.

### Transcriptomics

RNA from approximately 30 mg of mouse neocortical tissue was isolated using Direct-zol RNA Microprep (R2062, Zymo Research, Germany) according to manufacturer’s instructions. Transcriptome analysis was performed at BGI (China). After cDNA library preparation, 100 bp pair-ended sequencing was performed on a DNBSEQ platform with a depth of ~ 20 million read pairs per sample. Data were aligned to the mouse genome (GRCm38.p6) and differential gene expression was analyzed using DESeq2 [28]. Gene ontology analysis was performed using clusterProfiler [60]. Meaningful and highly significantly enriched GO categories were manually selected and depicted in Fig. 6C. The full expression data is presented in Additional file 2: Table S1.

### Monoclonal antibody generation from immunized Balb/c and C57BL/6J mice

Balb/c mice were immunized with 40 µg of OVA-coupled TDP-43 peptide #7 (aa. 153–191) together with 5 nmol CpG (TIB MOLBIOL, Germany) in 200 ul PBS and an equal volume of Incomplete Freund's adjuvant (IFA; Sigma, USA). Booster injections without IFA were given 8 and 13 weeks later. C57BL/6J mice from the pilot immunization cohort (Fig. 1B/C) were treated as described before but received an additional booster injection before splenectomy. Immune spleen cells from immunized Balb/c and C57BL/6J mice vaccinated with other epitopes (see Additional file 1: Fig. S1A) were collected three days (Balb/c mice) or seven days (C57BL/6J) after the final boost for fusion with P3X63Ag8.653 myeloma cells using standard procedures. Hybridoma supernatants were screened 10 days later in a flow cytometry assay (iQue, Intellicyt, Sartorius, Germany). Briefly, recombinant TDP-43-MBP-His_6_ fusion protein was captured to 3D-Aldehyde beads (PolyAN, Germany) and incubated for 90 min with hybridoma supernatant and Atto 488-coupled isotype-specific monoclonal rat anti-mouse IgG secondary antibodies. Antibody binding was analyzed using ForeCyt software (Sartorius, Germany). Clones were further validated and selected for up-scaling by sandwich ELISA. Briefly, Nunc MaxiSorp™ flat bottom 96-well plates were coated with either N-terminal (10782-2-AP, Proteintech) or C-terminal (12892-1-AP, Proteintech) binding TDP-43 antibody overnight at 4 °C. The next day, recombinant TDP-43-MBP-His_6_ was added in serial dilution for 2 h at RT, followed by a 2 h incubation with the hybridoma supernatants at RT. As a secondary antibody, the respective HRP-conjugated isotype-specific mouse IgG antibody (Helmholtz Center Munich) was added for 1 h at RT. Between each step, plates were washed three times with PBS supplemented with 0.05% Tween 20. Plates were measured as described before (see TDP-43 antibody ELISA) and supernatants with the best signal-to-noise ratio with both N-terminal and C-terminal TDP-43 capture antibodies were selected. Hybridoma cells from selected supernatants were subcloned at least twice by limiting dilution to obtain stable monoclonal cell lines (see Additional file 1: Fig. S1A).

### Recombinant protein expression and purification

#### *TDP-43-MBP-His*_*6*_

Human TDP-43-MBP-His_6_ was purified as described before [16] from plasmid pJ4M TDP-43-TEV-MBP-His_6_ (addgene #104480) [57]. Briefly, protein expression was performed in *E. coli* BL21-DE3 Rosetta 2 induced with 0.5 mM IPTG and grown overnight at 16 °C. Bacteria were resuspended in lysis buffer (20 mM Tris pH 8.0, 1 M NaCl, 10 mM imidazole, 10% glycerol, 4 mM β-mercaptoethanol and 1 µg/ml each of aprotinin, leupeptin hemisulfate, and pepstatin A) supplemented with 0.1 mg/ml RNase A and lysed using lysozyme and sonication. Next, the protein was purified by Ni–NTA agarose (Qiagen) and eluted with lysis buffer containing 300 mM imidazole. To separate monomeric TDP-43-MBP-His_6_ from protein aggregates and contaminants, a final size exclusion chromatography (SEC) (Hiload 16/600 Superdex 200 pg, GE Healthcare) purification step was performed in purification buffer (20 mM Tris pH 8.0, 300 mM NaCl, 5% glycerol supplemented with 2 mM TCEP). The purified protein was concentrated using Amicon ultra centrifugal filters, then flash frozen, and stored at −80 °C. To determine protein concentration, absorbance at 280 nm was measured using the respective extinction coefficient (ε) predicted by the ProtParam tool. Moreover, the A260/280 ratio was determined and found to be between 0.5–0.7. Prior to use in any downstream assays assessing phase separation, TDP-43-MBP-His_6_ aliquots were centrifuged (21,000 × g for 10 min at 4 °C) to remove preexisting protein precipitates.

#### *His*_*6*_*-TEV protease*

His_6_-TEV protease expression and purification was performed as described in [20].

### Fluorescent labeling of purified TDP-43 and monoclonal antibodies

TDP-43-MBP-His_6_ was labeled with Alexa Fluor 488 C5 maleimide (Thermo Fisher) and monoclonal TDP-43 antibodies were conjugated with DyLight™ 650 NHS Ester (Thermo Fisher), both at a low (~ 0.01–0.05) labelling efficiency to not interfere with condensate formation. Following manufacturer´s instructions, TDP-43-MBP-His_6_ was mixed with the Alexa Fluor reagent in a 100:1 or 20:1 protein:fluorescent dye molar ratio and kept in the dark for 2 h at RT. Monoclonal antibodies and DyLight dye were used in a 50:1 protein:fluorescent dye molar ratio and incubated for 1 h at RT protected from light. Excess dye was removed by consecutive washes with TDP-43 purification buffer (TDP-43-MBP-His_6_) or PBS (monoclonal antibodies) using Amicon ultra centrifugal filters (Merck, Germany) and protein concentrations were determined. Labeled proteins were used for confocal microscopy, aggregation, and phase separation assays, respectively. For flow-cytometry based uptake assays, TDP-43-MBP-His_6_ was labeled with pHrodo™ iFL Green STP Ester (Thermo Fisher) at a labeling efficiency of ~ 0.7–1.0. The labeling procedure was carried out as described above with a protein:fluorescent dye molar ratio of 1:1 and an incubation step of 1 h.

### In vitro aggregation and phase separation assays

#### Aggregation assay

In 1.5 ml low binding tubes (Eppendorf), 8 µM Alexa 488-labeled TDP-43-MBP-His_6_ was mixed with 4 µM unlabeled monoclonal antibody (isotype controls or TDP-43 antibodies, respectively) in aggregation buffer (50 mM Tris pH 8.0, 150 mM NaCl, 5% glycerol, 5% sucrose, 150 mM imidazole pH 8.0) supplemented with 1 × protease inhibitor (Sigma) and incubated with 100 µg/ml His_6_-TEV protease. Samples were constantly agitated at 1000 rpm for 30 min at RT and subsequently transferred into a 384-well imaging plate (781900, Greiner Bio-One, Germany), which was tightly sealed to avoid any evaporation. Samples were incubated for 48 h at RT and imaged using a LSM710 confocal microscope with a Plan-Apochromat 10x/0.45 M27 objective. Image acquisition was performed with a two-line averaging and at a pixel resolution of 1024 × 1024 (Carl Zeiss).

#### Sedimentation assay

1 µM TDP-43-MBP-His_6_ was mixed with 0.5 µM unlabeled monoclonal antibody (isotype controls or TDP-43 antibodies, respectively) in Hepes buffer (20 mM Hepes pH 7.5, 150 mM NaCl, 1 mM DTT). To remove the MBP-His_6_ solubilization tag and trigger liquid–liquid phase separation (LLPS), 20 µg/ml His_6_-TEV protease was added. The resulting condensates were pelleted by centrifugation (21,000× *g* for 15 min at 4 °C) after 2 h of incubation at 30 °C. Afterwards, equal amounts of supernatant (S) and condensate (C) fractions were loaded onto 10% SDS-PAGE gels, transferred to Nitrocellulose membranes and TDP-43 was detected by immunoblotting using an extreme C-terminal anti-TDP-43 antibody (epitope aa. 405–414, TIP-TD-P09, Cosmo Bio, USA). Quantification of the chemiluminescence signals in each lane was performed using an Amersham ImageQuant™ 800 imaging system (Cytiva, USA) and GelAnalyzer software (version 19.1). Ratios of the supernatant (S) and the total [supernatant (S) + condensate (C)] signals are represented and statistically analyzed.

#### Microscopic condensate assay

10% Pluronics F-127 solution was used to treat uncoated µ-Slide 18 Well—Flat chambers (81821, Ibidi, Germany) for 1 h at RT. Afterwards, wells were washed five times with MilliQ water. Prior to setting up the condensate formation reaction, the buffer of the purified Alexa 488-labeled TDP-43-MBP-His_6_ preparation was exchanged to Hepes buffer (20 mM Hepes pH 7.5, 150 mM NaCl, 1 mM DTT). 7 µM Alexa 488-coupled TDP-43-MBP-His_6_ was mixed with 3.5 µM DyLight 650-labeled monoclonal antibody (isotype controls or TDP-43 antibodies, respectively) in low binding tubes and 100 µg/ml His_6_-TEV protease was added to the reaction. Samples were transferred to µ-Slide 18 Well—Flat chambers and imaged using a Zeiss LSM710 confocal microscope after 30–60 min. For confocal microscopy, a Plan-Apochromat 63x/1.40 Oil DIC M27 objective was used and images were captured with two-line averaging at 2048 × 2048 pixel resolution.

To analyze fusion events of antibody-treated and TEV protease-cleaved TDP-43, condensates were formed in 18-well ibiTreat slides (81826, Ibidi) using 20 µM of Alexa 488-labeled TDP-43-MBP-His_6_, 10 µM of unlabeled monoclonal antibodies (isotype control IgG2c, 36C5 or 36C10, respectively) and 100 µg/ml His_6_-TEV protease in Hepes buffer. After an incubation step of 10 min at RT, condensates were imaged in 5 s intervals within a time window of up to 45 min after cleavage. An inverted spinning disc microscope (Visiscope 5 Elements, Visitron Systems GmbH, Germany), built on a Nikon Ti2 stand equipped with a confocal spinning disc (CSU-W1; Yokogawa, Japan) with 50 µm pinhole diameter and a 60x/1.2 NA water immersion objective was used for acquisition. Imaging was performed using the 488 nm laser line for excitation and 525/50 bandpass filter (Chroma) for collecting the fluorescence emission. Images were captured using sCMOS camera (Prime BSI, Photometrics) and a frame of 1033 × 1033 pixels in 12-bit acquisition mode.

### TDP-43 uptake assay

SH-SY5Y cells were cultured in DMEM/F12 (1:1) medium + L-Glutamine + 15 mM Hepes (Gibco) supplemented with 10% fetal bovine serum, until they reached a confluency of ~ 80%. Cells were then harvested and seeded in a 24-well plate (5 × 10^4^ cells/well). The following day, recombinant pHrodo Green-labeled TDP-43-MBP-His_6_ was used to prepare 10 µM of aggregates during an incubation step of 2 h (see *Aggregation assay*). Subsequently, 10 µM of single mAbs (isotype controls IgG1, IgG2c, 30D3, 31E9, 36C5 or 36C10) or equivalent amounts of PBS were added individually to the TDP-43 aggregates and incubated for 30 min at RT. Afterwards, media of SH-SY5Y cells were aspirated completely and 500 μl of fresh media containing 500 nM TDP-43 aggregate/mAb mix or 500 nM monomeric TDP-43-MBP-His_6_ were added on cells for ~ 20–24 h at 37 °C and 5% CO_2_. The day after, cells were washed twice and harvested for flow cytometry analysis. Flow cytometry experiments were performed on a BD FACSVerse™ instrument (BD Biosciences, USA) and analyzed using FlowJo software (version 10.6.2, Treestar). In brief, singlets were detected using FSC-A versus FSC-H parameters. Live cells were identified as SYTOX™ Blue^−^ (negative) (Thermo Fisher) cells out of the single cell population. Lastly, the relative fluorescence of FITC in live single cells was analyzed. For each condition tested, at least 1800 live cells were recorded.

### Data visualization and statistical analysis

Experimenters were blinded to genotype during immunization study and all subsequent immunoassays in which samples were distributed randomly. Animal experiments (immunizations and blood collection) were carried out in random order. Data were visualized and statistically analyzed using R (version 3.6.1) with RStudio. Assumptions for parametric tests were assessed by Shapiro–Wilk test/Q-Q plots and Levene’s test. Longitudinal changes of body weights were analyzed by two- or three-way repeated measures ANOVA. One-way ANOVA followed by pairwise t-test with Benjamini–Hochberg correction was used as parametric test to statistically compare more than two groups. Kruskal–Wallis test and subsequent pairwise Wilcoxon Rank Sum Tests with Benjamini–Hochberg correction were used as non-parametric tests. Individual datapoints are generally displayed as scatterplots plus bar graphs [mean + standard deviation (SD)] or box plots (box indicates lower quartile, median and upper quartile, whiskers extend to the most extreme data point within 1.5 × the interquartile range of the box). *P*-values within graphs are reported as follows: * denotes *p* ≤ 0.05, **p ≤ 0.01, ***p ≤ 0.001, ****p ≤ 0.0001.

## Results

### Immunogenicity and safety greatly vary between TDP-43 peptide epitopes

To determine which epitopes of TDP-43 induce sufficient antibody titers to slow disease progression in a TDP-43 mouse model, we selected 14 peptide antigens, collectively covering all domains and 2/3 of sequence of human TDP-43 (Fig. 1A and Additional file 1: S1A), based on predicted antigenicity. Within long, continuous, predicted antigenic stretches, we chose peptides of up to ~ 40 amino acids in length (peptide 7, 10 and 12). Apart from peptides 5, 7, 8, 11 and 12, all epitopes are fully conserved between human and mouse TDP-43. In addition, we designed peptide 15 to include phosphorylated serines 409/410 because phosphorylation at these sites is highly disease-specific [18]. To increase immunogenicity, we conjugated all peptides to ovalbumin (OVA). After three immunizations of wild-type C57BL/6J mice with the OVA conjugates, antibody responses were measured in an indirect ELISA using recombinant full-length human TDP-43. Peptides 1, 3, 10, 12, and 14 elicited the strongest antibody response, while peptides 4,6, and 13 induced the lowest antibody titers (Fig. 1C). As expected, preimmune sera did not detect TDP-43 (Additional file 1: Fig. S1B). Notably, sera from mice immunized with the C-terminal phospho-peptide did not bind to non-phosphorylated recombinant TDP-43, but strongly reacted with the cognate phospho-peptide by indirect ELISA (Fig. 1C, right panel). Immunization with all peptides was well tolerated without apparent side effects in wild-type C57BL/6J mice.

To identify therapeutically relevant epitopes, we vaccinated rNLS8 mice that develop key features of human ALS/FTD pathology, such as cytoplasmic aggregation and nuclear clearance of TDP-43 as well as progredient motor deficits upon induction of the hTDP-43ΔNLS transgene following doxycycline withdrawal [56]. Due to the large number of highly immunogenic antigens, bigenic mice (hereafter called rNLS8) were immunized with five pools of antigens (Fig. 1D): we used the two peptides with the highest antigenicity (Fig. 1C) from either the N-terminus (peptides 1 and 3), RRM1 (peptides 5 and 7), RRM2 (peptides 8 and 9) or the glycine-rich low-complexity domain (peptides 10 and 12) as well as the antigen harboring the disease-specific phosphorylation site (peptide 15). Mock-immunized bigenic animals (TDP-PBS) served as controls to evaluate therapeutic benefits. Potential side effects mediated by active immunization in a non-diseased context were monitored by treatment of monogenic control animals (not expressing the TDP-43ΔNLS transgene upon doxycycline withdrawal) with a mixture of all peptide antigens (peptides 1, 3, 5, 7, 8, 9, 10, 12, 15). Based on previous results [63], all animals were vaccinated five times prior to, and once after, transgene induction to trigger high antibody titers (Fig. 1E). All rNLS8 mice developed progressive weight loss and muscle weakness and were sacrificed 21 days after doxycycline withdrawal to facilitate direct comparison of treatment effects on pathology (see below).

In contrast to the immunization in wild-type C57BL/6J mice, combined peptides 1 + 3 caused acute lethality in rNLS8 mice with a mixed C57BL/6J × C3H/HeJ background even prior to transgene expression. Seven of the ten mice immunized with the pooled peptides 1 and 3 unexpectedly died or had to be sacrificed shortly after the second or third immunization and we thus stopped further boosting the remaining three animals (Fig. 1F, dashed line). All other peptides were equally well tolerated in the rNLS8 and control mice. To address the role of the different genetic backgrounds for the unexpected toxicity, we conducted two immunizations with the antigen pools from Fig. 1D in C57BL/6J, C3H/HeOuJ, and CD-1 mice (Additional file 1: Fig. S2A-D). The antibody titers were comparable between the three lines (Additional file 1: Fig. S2B). Surprisingly, only one of six CD-1 and none of the C3H/HeOuJ or C57BL/6J mice receiving peptides 1 + 3 developed symptoms requiring euthanization. Hence, immunization with TDP-43 peptides shows strikingly different side effects in different mouse strains, which may be attributed to a loss-of-function mutation in the Tlr4 receptor found in C3H/HeJ mice but not the otherwise genetically very similar C3H/HeOuJ line [8]. Nevertheless, our data establish several highly antigenic and potentially safe epitopes for active TDP-43 immunotherapy.

### Epitopes in the glycine-rich domain of TDP-43 induce a strong antibody response

**Fig. 2:**
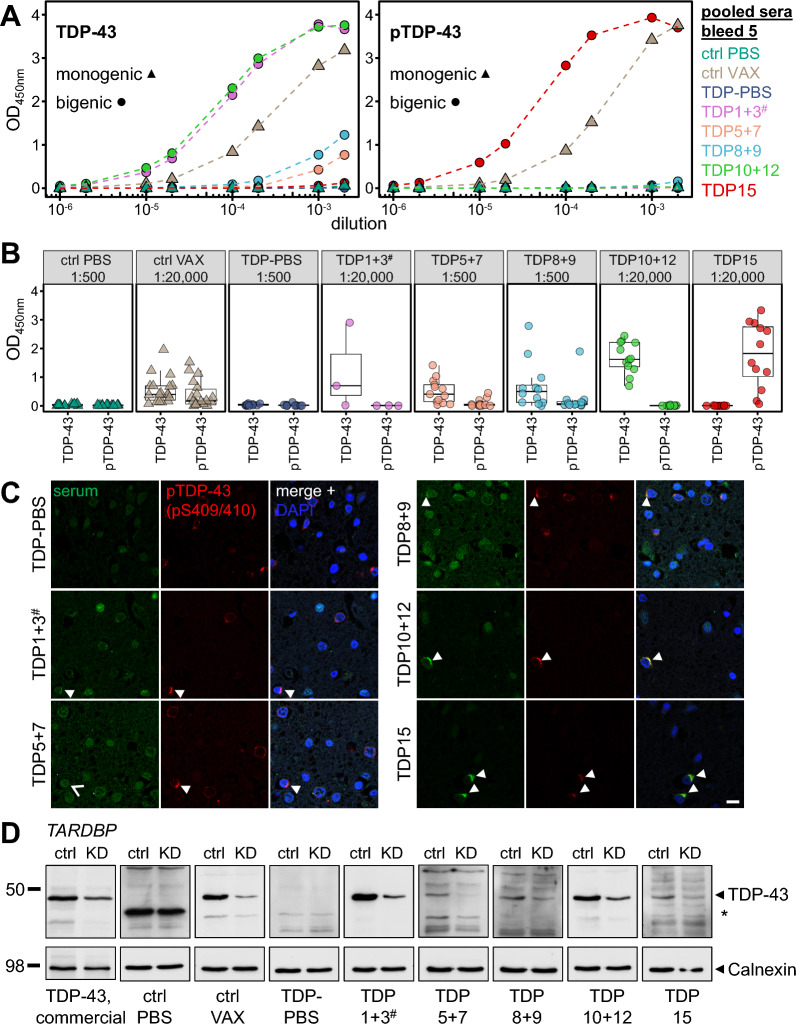
C-terminal TDP-43 peptides elicit high-titer antibodies detecting patient TDP-43 aggregates. TDP-43-specific antibody responses in sera from mice receiving five immunizations (expect TDP1 + 3 with 1–3 immunizations) were analyzed by ELISA, immunofluorescence stainings, and immunoblot. **A** A dilution series of pooled sera was analyzed by ELISA against recombinant full-length TDP-43 (left panel) or pTDP-43 peptide (pS409/410, right panel). Background-corrected mean OD450 was measured in duplicates. Triangular shapes indicate monogenic animals, circles represent rNLS8 mice (labeling consistent throughout the following figures). **B** Antibody response in antisera from individual mice was measured as in (**A**) in single dilutions (1:500 or 1:20,000) to analyze inter-animal variability. Control (ctrl) PBS n = 17, ctrl VAX n = 18, TDP-PBS n = 10, TDP1 + 3 n = 3, TDP5 + 7 n = 13, TDP8 + 9 n = 13, TDP10 + 12 n = 12, and TDP15 n = 12. Note that the number of immunizations in TDP1 + 3 animals (1, 2 or 3) correlates with the resulting titer. **C** Double immunofluorescence stainings of pooled antisera and phosphorylated TDP-43 (pS409/410) were performed on frontal cortex sections of a sporadic FTLD case. Representative images are shown. Arrowheads indicate strong (TDP10 + 12, TDP15) or weak (TDP1 + 3, TDP8 + 9) labeling of pTDP-43 inclusions with antisera. TDP5 + 7 antiserum failed to detect pTDP-43-positive inclusions (open arrowhead). Images from a healthy control are shown in Fig. S3A. Scale bar = 10 µm. **D** Immunoblotting of HEK293 cell lysates of doxycycline-inducible *TARDBP* knockdown (KD) and control (ctrl) using a commercial TDP-43 antibody (left lane) or pooled antisera. Asterisk denotes a prominent non-specific band detected with the ctrl PBS serum. Calnexin was used as a loading control

Next, we analyzed the antibody response in immunized rNLS8 mice by ELISA, immunofluorescence and immunoblots. Using an ELISA against recombinant full-length TDP-43, dilution series of pooled sera from five-times immunized animals revealed the strongest immune response in mice vaccinated with peptides 10 + 12. The three surviving mice from the TDP1 + 3 group had a similarly strong response, although they only received one, two or three immunizations (Fig. 2A, left panel). Peptide pools 5 + 7 and 8 + 9, which targeted RRM1 and RRM2, respectively, induced weaker antibody responses. Antisera from animals immunized with the phospho-peptide (TDP15) did not detect unphosphorylated TDP-43 expressed in *E. coli* (Fig. 2A, left panel), but strongly reacted with the cognate phospho-peptide (Fig. 2A, right panel). Antisera from monogenic control mice receiving the antigen mix including the phospho-peptide (#15) also detected the phospho-peptide by ELISA, albeit at a lower level.

Due to the lack of dilutional linearity in the ELISA, we compared the antibody response of individual animals within each group using a fixed dilution of the antisera. As in the pooled analysis (Fig. 2A), the antibody response was most robust in the TDP10 + 12 and TDP15 animals (Fig. 2B). Of note, the three doses of peptides 1 + 3 were highly immunogenic in the surviving animal consistent with results in three inbred mouse strains (Additional file 1: Fig. S2B). To analyze the kinetics of the antibody response, we compared the antibody response for pooled sera from all groups over time (Additional file 1: Fig. S2E). In contrast to our anti-GA immunization [63], anti-TDP-43 titers plateaued already two weeks after the second immunization, suggesting that preselecting highly immunogenic epitopes within TDP-43 strongly accelerated the response.

We next asked whether the antisera could detect disease-associated TDP-43 inclusions in human patient sections. Indeed, antisera of TDP10 + 12 and TDP15 mice strongly stained the characteristic cytoplasmic pTDP-43-positive inclusions in brain sections of an FTLD patient which are absent in a healthy control, with TDP15 antisera showing almost no affinity for nuclear TDP-43 compared to TDP10 + 12 (Fig. 2C, Additional file 1: S3A). TDP1 + 3 and, to a lesser extent, TDP8 + 9 antisera still detected some pTDP-43 inclusions as well as nuclear TDP-43. At the same dilution, antisera from TDP5 + 7 immunized animals failed to stain cytoplasmic aggregates. Co-staining of human tissue with commercial pan-TDP-43 antibody further confirmed specificity of the antisera (Additional file 1: Fig. S3B).

To test whether the strong ELISA and immunofluorescence signal depend on the native TDP-43 conformation and to rule out poor accessibility of the certain epitopes in native TDP-43, we validated the specificity of antisera using denatured TDP-43 in cellular lysates of a CRISPR interference (CRISPRi) mediated inducible *TARDBP* knockdown in stably transfected HEK293 cells. Pooled antisera specifically detected a ~ 43 kDa band of endogenous human TDP-43 that was reduced upon doxycycline-induced knockdown of *TARDBP* transcription (Fig. 2D). As expected, the TDP15 antisera showed a very low signal in the absence of pathological TDP-43 phosphorylation in HEK293 cells. Consistent with the ELISA data, TDP1 + 3 and TDP10 + 12 antisera showed the strongest signals and were highly TDP-43-specific.

Taken together, antigens derived from the glycine-rich domain (peptides 10 + 12 and 15) reliably induced high-titer antibodies and detected disease-associated cytoplasmic TDP-43 inclusions.

### Immunization with the C-terminal pS409/410 epitope reduces NfL levels in rNLS8 mice without slowing the rapid body weight loss

**Fig. 3:**
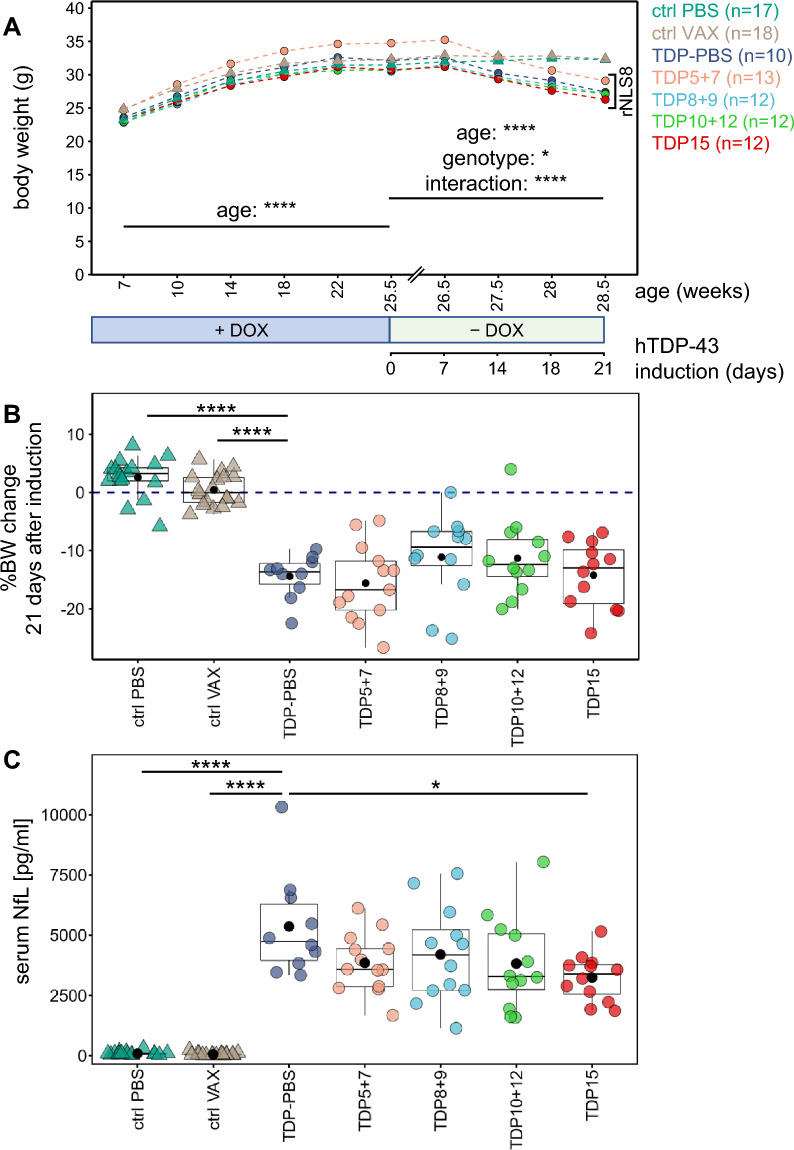
TDP-43 active immunization does not prevent weight loss, but the pTDP-43 antigen moderately lowers NfL. Body weight and NfL levels were analyzed in immunized control and rNLS8 mice. **A** Development of body weight in control and rNLS8 mice before and after transgene induction using doxycycline withdrawal. Mean values are plotted group-wise, *n* as indicated. The longitudinal effects of treatment (peptide immunization vs. no immunization), genotype (control vs. rNLS8), age as well as their interactions were analyzed using a three-way repeated measures ANOVA. On doxycycline chow, only age had a statistically significant effect on the body weight (*p* = 1.99*10^–102^), but not the treatment (*p* = 0.74). After transgene induction, genotype (*p* = 0.018), age (*p* = 2.34*10^–26^), and their interaction (*p* = 2.20*10^–26^), but not treatment (*p* = 0.901), had a statistically significant effect on the body weight. **B** Comparison of the percentage body weight (BW) loss from transgene induction (25.5 weeks of age) to end-stage (28.5 weeks of age) against the TDP-PBS group using pairwise t-test with Benjamini–Hochberg correction revealed a statistically significant difference for both monogenic groups (ctrl PBS vs. TDP-PBS: *p* = 1.47*10^–11^, ctrl VAX vs. TDP-PBS: *p* = 6.38*10^–10^) only. Group means are indicated as black dots. **C** Neurofilament light chain (NfL) levels in the serum of end-stage mice were quantified using the Simoa® platform. Comparisons against TDP-PBS mice using pairwise Wilcoxon test with Benjamini–Hochberg correction revealed a statistically significant difference in NfL levels for ctrl PBS (*p* = 8.3*10^–7^), ctrl VAX (*p* = 8.3*10^–7^), and TDP15 immunized mice (*p* = 0.013). Mean values are indicated as black dots

Since disease-relevant phenotypes progress rapidly in aged rNLS8 mice [51], we sacrificed all mice 21 days after transgene induction, when the first animals reached humane endpoint criteria (mostly weight loss), allowing us to compare the pathology and progression at a fixed time point (Fig. 1E). As body weight loss is a cardinal symptom of ALS patients and the rNLS8 mouse model [56], we monitored body weight of all immunized mice to assess the efficacy and potential adverse effects of the immunization (Fig. 3A). While there was no significant effect of vaccination on body weight prior to transgene expression, all rNLS8 mice lost weight ~ 14 days after transgene induction regardless of the immunization regimen (Fig. 3A/B).

Neurofilament levels in blood and CSF are emerging as a powerful biomarker for detecting neuroaxonal damage in mouse models and patients affected by ALS [22]. Thus, we measured neurofilament light chain (NfL) levels in serum collected from mice at the time of sacrifice (bleed 6). We found that serum NfL was strongly elevated in rNLS8 mice compared to the monogenic controls (Fig. 3C). Interestingly, immunization with the highly immunogenic peptide 15 significantly reduced serum NfL in rNLS8 mice compared to the TDP-PBS control, suggesting that targeting the disease-specific phospho-epitope by active immunization might have beneficial effects on TDP-43-mediated disease progression.

### None of the peptide antigens reduces pTDP-43 pathology or gliosis in immunized mice

**Fig. 4:**
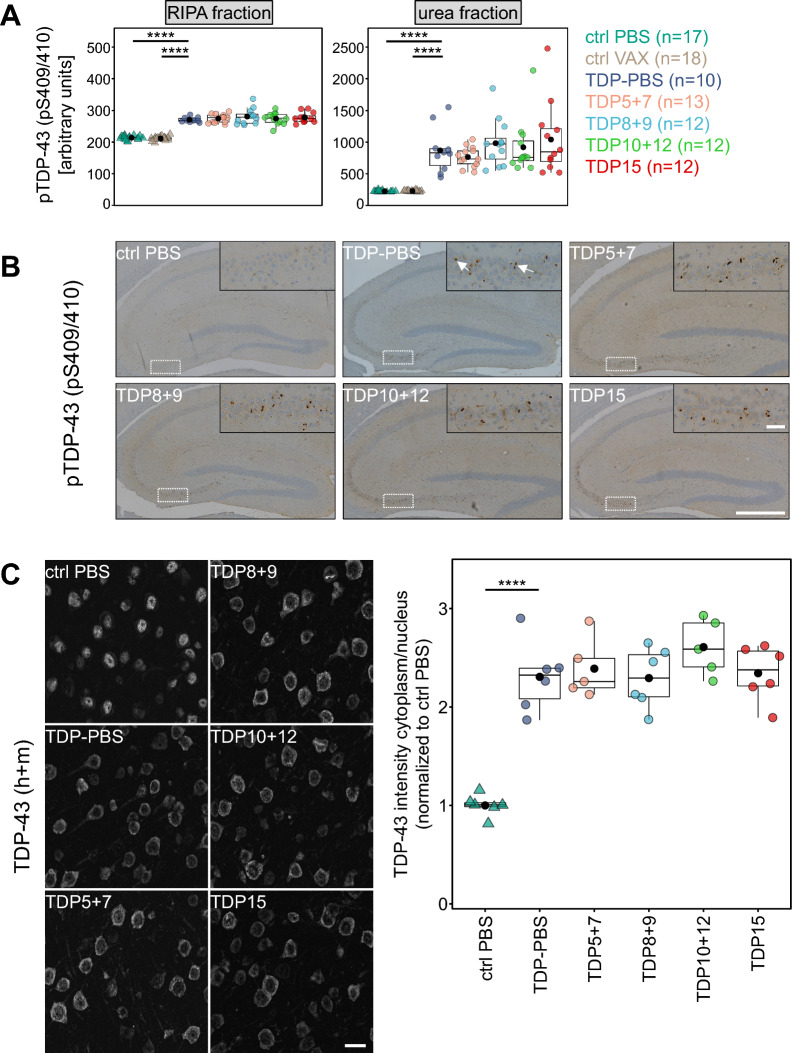
Active immunization does not affect pTDP-43 levels and TDP-43 cytoplasmic-to-nuclear distribution in rNLS8 mice. Cerebral TDP-43 pathology was analyzed by ELISA, immunohistochemistry, and immunofluorescence stainings in vaccinated rNLS8 mice. **A** Phospho-TDP-43 (pS409/410) levels were measured in RIPA and urea fractions from neocortex. Only monogenic animals showed statistically significant differences in RIPA lysates (ctrl PBS: *p* = 7.63*10^–5^, ctrl VAX: *p* = 7.63*10^–5^) and urea lysates (ctrl PBS: *p* = 7.70*10^–5^, ctrl VAX: *p* = 7.70*10^–5^) when compared to TDP-PBS group by pairwise Wilcoxon test with Benjamini–Hochberg correction. Mean values are plotted as black dots. **B** Immunohistochemical staining in the hippocampal region using an antibody against pS409/410 TDP-43. Representative images for each group are shown. Magnified insets are displayed on the top right corner and their position in the overview is depicted by dashed rectangles. Arrows in the inset denote characteristic pTDP-43 inclusions in the CA3 region. Scale bar overview = 500 µm, scale bar inset = 50 µm. C: Immunofluorescence staining of total TDP-43 (human + mouse) in the frontal neocortex. Representative images for each group are depicted in the left panel (Scale bar = 20 µm). Note that control PBS images were taken at a higher gain to compensate for the lower levels of endogenous TDP-43. Right panel shows quantification of the ratios of the cytoplasmic-to-nuclear TDP-43 intensities (after normalization to the ctrl PBS group). Mean values are plotted as black dots. Pairwise t-test with Benjamini–Hochberg correction indicated a statistically significant difference between monogenic ctrl PBS and bigenic TDP-PBS animals (*p* = 3.32*10^–5^). Control (ctrl) PBS n = 6, TDP-PBS n = 6, TDP5 + 7 n = 5, TDP8 + 9 n = 6, TDP10 + 12 n = 5, and TDP15 n = 6

To further investigate the potential benefits of TDP-43 peptide vaccination in rNLS8 mice, we analyzed disease-associated pS409/410 TDP-43 levels and neuroinflammation in treated animals. Using a previously established immunoassay, we quantified pTDP-43 in the cerebral cortex (Fig. 4A). This assay showed a significant accumulation of pTDP-43 in the rNLS8 mice compared to controls. As expected, pTDP-43 was strongly enriched in the urea-soluble fraction compared to the RIPA-soluble fraction. However, pTDP-43 levels did not differ between the rNLS8 groups, indicating that even high antibody titers in serum cannot prevent brain pTDP-43 accumulation in rNLS8 mice.

These findings were confirmed by pTDP-43 immunohistochemistry, which visualized characteristic pTDP-43 inclusions in the CA3 area of the hippocampus, which is like the neocortex considerably affected by TDP-43 pathology three weeks after transgene induction (Fig. 4B, arrows). In addition, we analyzed subcellular TDP-43 distribution to complement the pTDP-43 data. Immunofluorescence stainings using a pan-TDP-43 antibody (detecting both human and mouse TDP-43) revealed pronounced cytoplasmic TDP-43 signal in bigenic animals compared to a predominant nuclear localization of endogenous TDP-43 in monogenic mice (Fig. 4C). The ratio of cytoplasmic-to-nuclear TDP-43 was not affected in any of the treatment groups.

**Fig. 5:**
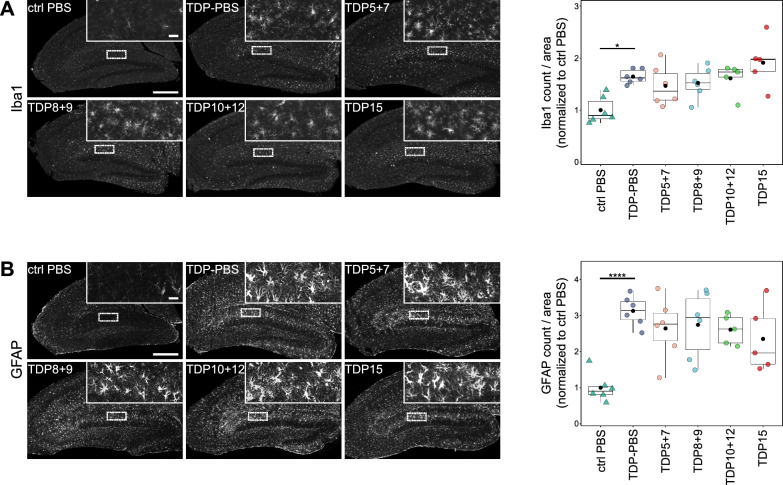
Profound micro- and astrogliosis in the rNLS8 model are unchanged upon vaccination with TDP-43 peptides. Using an automated staining and analysis pipeline, microglia (Iba1) and astrocyte (GFAP) signals were quantified in hippocampal regions. Representative immunofluorescence images of Iba1 (**A**) or GFAP (**B**)-stained hippocampal regions. Magnified insets are shown on the top right corner and their position in the overview is indicated by dashed rectangles. Scale bar overview = 500 µm, scale bar inset = 50 µm. Right panels show automated quantification of the Iba1 (**A**) and GFAP (**B**) cell count per area, followed by normalization to the ctrl PBS group. Mean values are plotted as black dots. Pairwise Wilcoxon test with Benjamini–Hochberg correction revealed an increased Iba1 count in the TDP-PBS group when compared to ctrl PBS animals (*p* = 0.011). GFAP counts were analyzed using pairwise t-test with Benjamini–Hochberg correction and were statistically different between TDP-PBS and ctrl PBS mice (*p* = 2.04*10^–5^). None of the vaccinated groups are significantly different from TDP-PBS. Control (ctrl) PBS n = 6, TDP-PBS n = 6, TDP5 + 7 n = 6, TDP8 + 9 n = 6, TDP10 + 12 n = 5, and TDP15 n = 5

Moreover, GFAP and Iba1 immunofluorescence revealed widespread early microgliosis (Fig. 5A) and astrogliosis in rNLS8 mice compared to controls (Fig. 5B). However, immunization with neither of the antigens markedly affected astrocyte or microglia activation as indicated by their comparable abundance and cellular morphology.

### Immunization against C-terminal epitopes slightly rescues the gene expression changes in rNLS8 mice

**Fig. 6:**
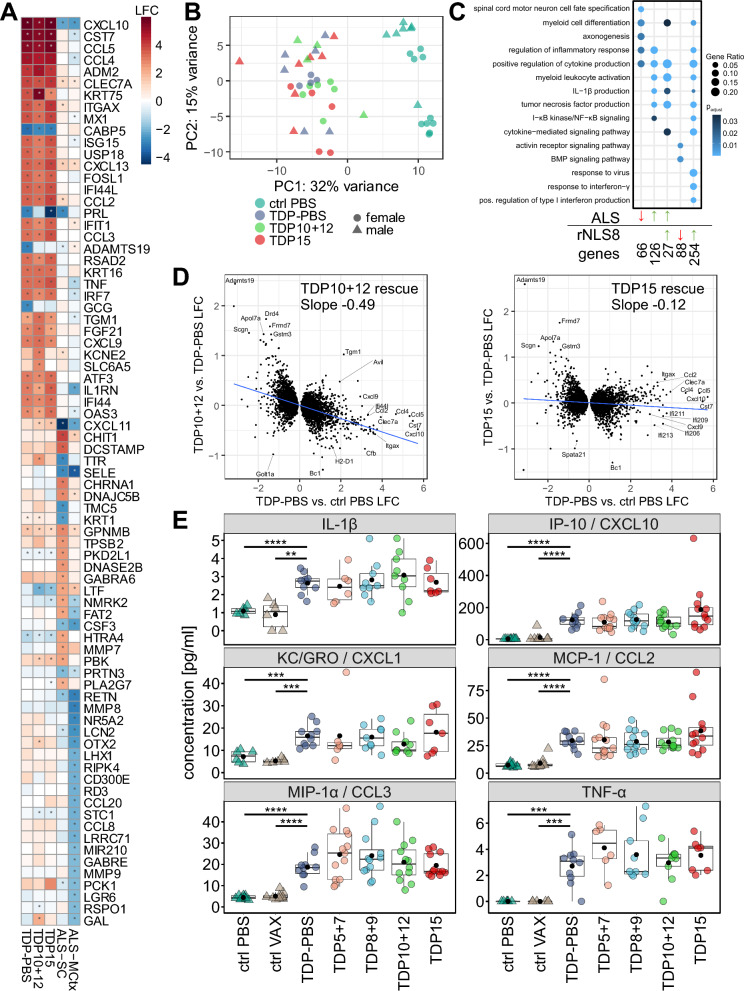
Widespread transcriptional changes in rNLS8 mice are modestly rescued by immunization targeting the glycine-rich region. **A** Differentially expressed genes from rNLS8 mice (TDP-PBS, TDP10 + 12, TDP15) compared to controls (ctrl PBS) and motor cortex (MCtx) and spinal cord (SC) of sporadic ALS patients compared to no-disease controls (dataset from [25]). From each group the 25 genes with largest absolute log2 fold change (LFC) also reaching statistical significance (indicated by asterisk) are depicted. Color code denotes LFC. **B** Principal component analysis (PCA) of transcriptomes separates animals based on genotype (control vs. rNLS8), but not on therapeutic intervention or gender. **C** Gene ontology analysis of differentially expressed genes (cutoff |LFC|> 1) in ALS spinal cord and TDP-PBS mice, each both compared to non-diseased controls. Manual selection of categories with > 4 genes. Full list is shown in Additional file 3: Table S2. **D** Fold-change of genes differentially expressed in TDP-PBS vs. control mice plotted against the fold-change between vaccinated and PBS-treated rNLS8 animals. Blue line shows linear regression. Pearson's product-moment correlation shows partial rescue of gene expression (p < 2*10^–16^ for both, correlation coefficient −0.49 and −0.12 as indicated). **E** Cytokine levels determined by multiplex assay from cortical tissue show inflammatory response in rNLS8 animals. Pairwise t-test with Benjamini–Hochberg correction revealed statistically significant differences between ctrl (monogenic) and bigenic TDP-PBS animals: IL-1β: ctrl PBS *p* = 2.57*10^–5^, ctrl VAX *p* = 0.004; IP-10: ctrl PBS *p* = 6.26*10^–5^, ctrl VAX: *p* = 6.26*10^–5^; KC/GRO: ctrl PBS *p* = 9.17*10^–4^, ctrl VAX *p* = 2.13*10^–4^; MCP-1: ctrl PBS *p* = 7.98*10^–6^, ctrl VAX *p *= 7.98*10^–6^; MIP-1α: ctrl PBS *p* = 3.44*10^–5^, ctrl VAX *p* = 3.44*10^–5^; TNF-α: ctrl PBS *p* = 5.64*10^–4^, ctrl VAX *p* = 5.64*10^–4^. TNF-α and IL-1β: Control (ctrl) PBS n = 6, ctrl VAX n = 6, TDP-PBS n = 10, TDP5 + 7 n = 6, TDP8 + 9 n = 9, TDP10 + 12 n = 9, and TDP15 n = 7; IP-10, KC/GRO, MCP-1 and MIP-1α: Control (ctrl) PBS n = 10, ctrl VAX n = 10, TDP-PBS n = 10, TDP5 + 7 n = 13, TDP8 + 9 n = 12, TDP10 + 12 n = 12, and TDP15 n = 12. Non-affected cytokines are shown in Supplemental Fig. S4C

To identify functional correlates of reduced NfL levels in TDP15 mice, we investigated neocortical gene expression profiles using RNAseq. To this end, we analyzed all PBS-treated controls (ctrl PBS) and rNLS8 mice (TDP-PBS) as well as all rNLS8 animals vaccinated with promising peptides from the C-terminal domain (TDP10 + 12 and TDP15). Transgene induction in rNLS8 mice dramatically altered the transcriptome, dominated by a strong inflammatory signature, which is in line with our immunofluorescence stainings of GFAP and Iba1 (Fig. 5). The data correspond well to the previously reported signature of isolated early- and late-stage rNLS8 microglia (Additional file 1: Fig. S4A) [19]. Vaccinated and PBS-treated rNLS8 mice showed an overall similar gene expression pattern when compared to control littermates (Fig. 6A). Top upregulated genes in rNLS8 mice included *Cxcl10*, *Ccl4*, *Ccl5,* and *Cst7*. Principal component analysis clearly separated controls from all rNLS8 groups but showed no clustering among the rNLS8 treatment groups (Fig. 6B). Comparison to the TargetALS RNAseq dataset from ALS spinal cord and motor cortex revealed shared differentially expressed genes, although the overlap is modest (Fig. 6A/C, Additional file 1: Fig. S4B). Gene ontology analysis indicated that many pathways induced in ALS spinal cords are also upregulated in rNLS8 mice, including IL-1β, TNF-α and NFκB signaling (Fig. 6C). The rNLS8 model displayed a stronger enrichment of interferon-related genes (Fig. 6C). The top concordant hits between rNLS8 neocortex and ALS spinal cord are inflammation-related (Fig. 6C and Additional file 1: S4B). No individual gene was differentially expressed in TDP10 + 12 or TDP15 immunized rNLS8 animals compared to the PBS-treated rNLS8 animals after multiple testing correction. However, comparing the log-fold changes between vaccinated and control rNLS8 animals (TDP10 + 12 vs. TDP-PBS and TDP15 vs. TDP-PBS) to transgene induced changes (TDP-PBS vs. ctrl PBS) (Fig. 6D) revealed a significant inverse correlation, which was more pronounced for the animals immunized with peptides 10 + 12. This indicates a trend for a slight overall rescue of transcriptional alterations in immunized rNLS8 mice.

To confirm inflammatory phenotypes identified by the transcriptomics and to assess potential therapeutic benefits of the immunization at the protein level, we measured the levels of 19 pro-inflammatory markers and cytokines in cortical brain lysates using a multiplexed immunoassay. While some of the analytes fell below detection levels at the tested concentrations (IFN-γ, IL-10, IL-12p70, IL-2, IL-4), we identified IL-1β, IP-10 (CXCL10), KC/GRO (CXCL1), MCP-1 (CCL2), MIP-1α (CCL3), and TNF-α to be significantly upregulated in rNLS8 mice compared to monogenic control animals (Fig. 6E). KC/GRO (CXCL1) and CCL2 are known to be activated by IL-1β and TNF-α via NFκB signaling and activate monocytes/microglia, while IP10 (CXCL10) is a well-known target of interferons consistent with the transcriptome data (Fig. 6C). In contrast, several interleukins measured in the panel were not altered in rNLS8 mice (Fig. S4C/D). No immunization regimen significantly affected levels of any cytokine quantified. Interestingly, among the markers measured by immunoassay, only *Cxcl10*, *Ccl2, Ccl3* and *Tnf* were strongly upregulated at the transcriptomic level as well (Fig. S4D). The chemokine profile in rNLS8 mice is likely driven by IL-1β and TNF-α and is consistent with strong monocyte/microglia activation (Fig. 5A).

### Novel monoclonal antibodies targeting the RRM2 or the C-terminal glycine-rich region inhibit TDP-43 aggregation in vitro

**Fig. 7:**
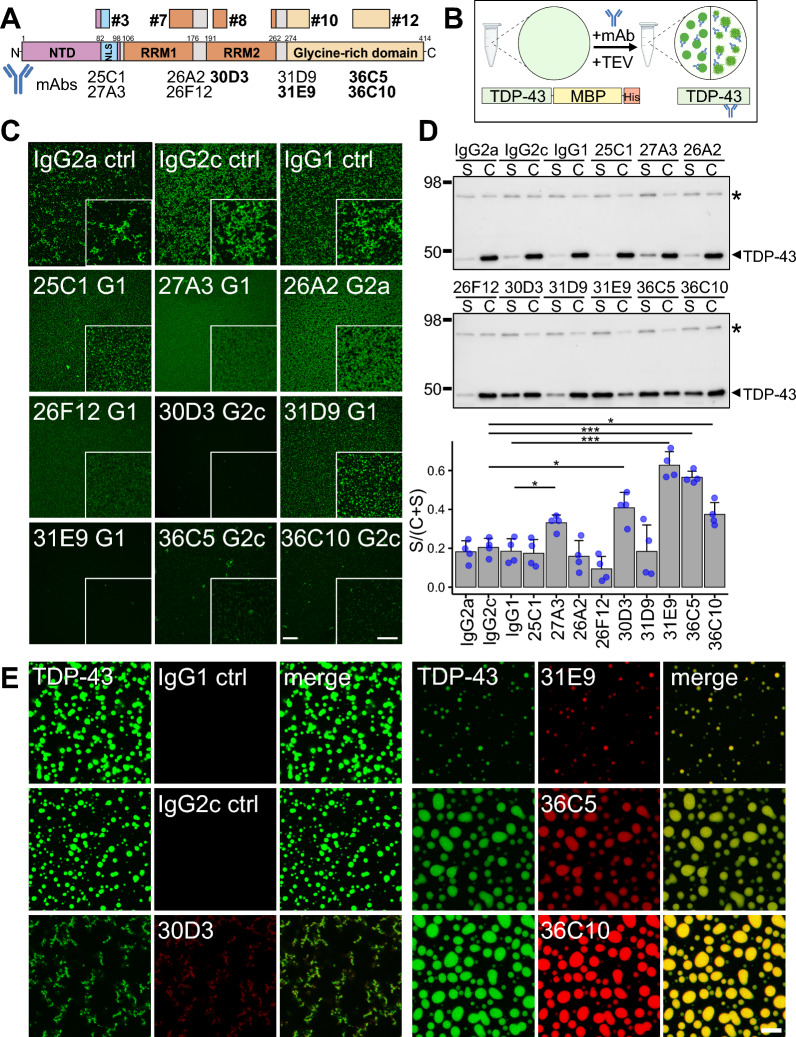
Antibodies targeting RRM2 and the glycine-rich region of TDP-43 suppress aggregation and LLPS in vitro*.* **A** Schematic overview of a panel of novel TDP-43 monoclonal antibodies (mAbs) raised from immunization in Fig. 1 as indicated. The most potent mAbs are highlighted in bold. **B** Aggregation and condensation assays using recombinant TDP-43-MBP-His_6_. Upon cleavage of the solubilization tag maltose-binding protein (MBP) using TEV protease, soluble TDP-43 forms aggregates (**C**) or condensates (**D**/**E**), depending on the buffer conditions. Condensates were either analyzed by pelleting (**D**) or by confocal microscopy (**E**). mAbs were added to soluble TDP-43-MBP-His_6_ together with TEV protease. **C** Representative images of Alexa 488-labeled and in vitro aggregated TDP-43, treated with the indicated mAb (molar ratio TDP-43-MBP-His_6_: mAb = 1:2). Images were taken 48 h after TEV cleavage. Clones 30D3 and 31E9 nearly completely prevented aggregation. Overview and magnified insets on the right bottom corner are shown. Scale bar overview = 100 µm, scale bar inset = 50 µm. **D** Sedimentation assay to analyze condensation of cleaved TDP-43-MBP-His_6_ in the presence of mAbs. Supernatant (S) and condensates (C) were separated by centrifugation 2 h after TEV cleavage. Representative immunoblots and quantification of S/(C + S) ratios (bar graphs indicate mean + SD) from four replicates are shown. An asterisk indicates the residual uncleaved TDP-43-MBP-His_6_, the arrowhead marks the cleaved TDP-43 band. mAbs were compared to their respective isotype controls using pairwise t-test with Benjamini–Hochberg correction for multiple testing: IgG2c vs. 30D3: *p* = 0.017; IgG2c vs. 36C5: *p* = 3.24*10^–4^; IgG2c vs. 36C10: *p* = 0.016; IgG1 vs. 27A3: *p* = 0.021; IgG1 vs. 31E9: *p* = 3.87*10^–4^. **E** Confocal images of Alexa 488-coupled TDP-43-MBP-His_6_ and DyLight 650-labeled mAbs show liquid–liquid phase separation of TDP-43 and co-partitioning of mAbs with TDP-43 condensates after TEV protease cleavage. Scale bar = 10 µm

To investigate the effects of antibodies induced by active vaccination in vitro, we generated a panel of monoclonal antibodies (mAbs) recognizing specific domains of TDP-43 by hybridoma generation using the spleens from immunized C57BL/6J mice (see Fig. 1B/C). Since we failed to generate mAbs from the spleens of C57BL/6J mice immunized with peptide 7, we generated mAbs by immunization of Balb/c mice with this peptide antigen. Using ELISA against recombinant TDP-43, we prescreened our 47 mAb clones and selected promising mAbs for the peptide epitopes 3, 7, 8, 10, and 12 (not shown) to characterize their effects on TDP-43 aggregation (Fig. 7A and Additional file 1: S1A).

To monitor TDP-43 aggregation in vitro, we used a recently established aggregate formation assay that relies on the cleavage of the solubilization tag MBP (maltose-binding protein) from a TDP-43-MBP-His_6_ fusion protein, followed by agitation in an aggregation-promoting buffer (Fig. 7B) [14, 16]. In the presence of isotype control mAbs (molar ratio antibody:TDP-43-MBP-His_6_ = 1:2), fluorescently-labeled TDP-43 readily formed aggregates that we visualized after 48 h (Fig. 6C, top panel). While clones 25C1, 27A3 (targeting peptide 3), 26A2, 26F12 (targeting peptide 7), and 31D9 (targeting peptide 10) had only moderate effects in suppressing TDP-43 aggregate formation, clones 30D3 (targeting peptide 8), 31E9 (targeting peptide 10), 36C5, and 36C10 (targeting peptide 12) strongly inhibited TDP-43 aggregate formation in this assay (Fig. 7C).

As TDP-43 aggregates are believed to arise from TDP-43 condensates that are formed by liquid–liquid phase separation (LLPS) and then undergo a liquid-to-solid state transition [3, 36, 57], we also assessed the capacity of the monoclonal antibodies to suppress TDP-43 condensate formation. Using a buffer containing physiological salt concentrations and no aggregation-promoting ingredients, TDP-43 rapidly undergoes LLPS after proteolytic removal of the MBP solubility tag [57]. To assess TDP-43 condensation in the presence of mAbs, we first used a sedimentation assay in which we separated the soluble fraction (S) and the condensate fraction (C) by centrifugation, followed by densitometric quantification of the TDP-43 signals in each fraction [16]. In line with the aggregation assay, clones 30D3, 31E9, 36C5, and 36C10 also significantly reduced TDP-43 condensate formation (Fig. 7D).

Next, we visualized TDP-43 condensates and assessed co-partitioning of mAbs into these condensates, by performing the condensation assay with fluorescently labeled TDP-43 and mAbs, and imaged condensates by confocal microscopy (Fig. 7E). As expected, isotype control mAbs did not co-localize with TDP-43 condensates, whereas 30D3 and 31E9 reduced condensate formation and co-partitioned into the remaining condensates. However, the 30D3-treated condensates appeared to have an amorphous, less liquid-like phenotype. In contrast, 36C5 and 36C10 increased the TDP-43 condensate size with prominent co-partitioning of both antibodies, suggesting that TDP-43 condensates become more fluid in the presence of these mAbs that both target an internal epitope of the glycine-rich domain. Fusion events observed by live imaging indeed indicated that condensates are larger and have more fluid-like character in the presence of antibodies 36C5 and 36C10 (Additional file 1: Fig. S5A). However, condensates fused relatively slowly and did not fully relax into perfectly round spheres, suggesting they are not ideal liquid-like droplets. Of interest, these four antibodies also showed the highest affinity among our pool of TDP-43 mAbs (Additional file 1: Fig. S5B). The comparable affinity among these four antibodies suggests that the exact epitopes drive the differential effect on condensate morphology in Fig. 7E. As expected, these four mAbs stained nuclear TDP-43 and cytoplasmic aggregates in FTLD brains (Additional file 1: Fig. S5C). Together, our data suggest that TDP-43 peptides 8, 10, and 12 can induce potent antibodies capable of inhibiting TDP-43 phase separation and aggregation in multiple in vitro assays.

### mAbs targeting the C-terminal glycine-rich region inhibit cellular uptake of TDP-43 aggregates

Since inhibition of cell-to-cell transmission could slow the progression in TDP-43 proteinopathies, we tested how the mAbs affect cellular uptake of TDP-43 aggregates.

**Fig. 8:**
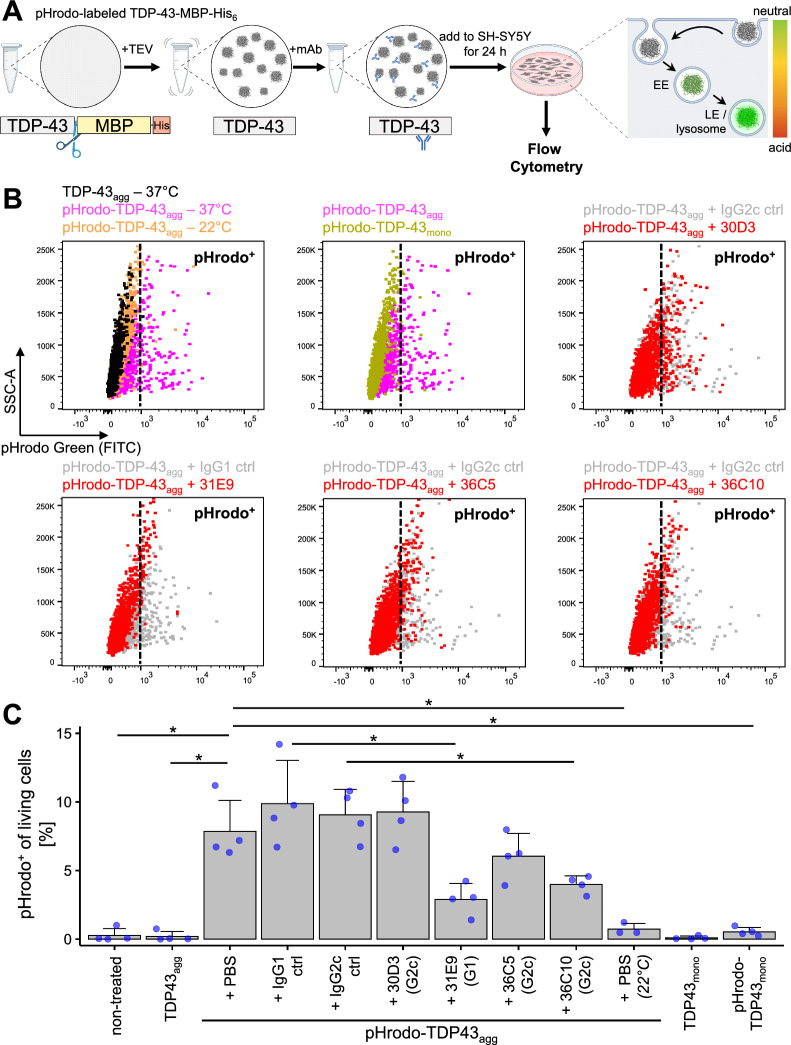
TDP-43 mAbs suppress cellular uptake of recombinant TDP-43 aggregates. **A** Overview of TDP-43 uptake assay. Aggregation of pHrodo Green-labeled TDP-43-MBP-His_6_ was induced by TEV protease cleavage and thorough shaking for 30 min, followed by a 2 h incubation step at room temperature. Preformed aggregates were then incubated with mAbs for 30 min before addition to SH-SY5Y cells. Upon cellular uptake via endocytosis, the pHrodo-tagged aggregates show increased fluorescence in acidic compartments, such as early endosomes (EE), and, even more so, in late endosomes (LE) or lysosomes. After 24 h of incubation, pHrodo fluorescence was analyzed by flow cytometry in the FITC channel. **B** Representative flow cytometry dot plots showing uptake of pHrodo-TDP-43_agg_ in combination with different control or TDP-43 specific mAbs in SH-SY5Y cells. Data areshown as relative fluorescence in the FITC channel (x-axis) and side scatter (SSC-A, y-axis). Relevant comparisons are overlaid. Cells were pregated to identify live singlets (not shown). Vertical dotted lines indicate gating for pHrodo^+^ live singlets. **C** Quantification of TDP-43 aggregate uptake in SH-SY5Y cells, measured as percentage of pHrodo-positive cells of total live single cells. Bar graphs represent mean + SD from n = 3–4 independent experiments. The following comparisons revealed statistically significant differences after pairwise t-test with Benjamini–Hochberg correction: pHrodo-TDP-43_agg_ + IgG1 ctrl vs. pHrodo-TDP-43_agg_ + 31E9 (G1): *p* = 0.037; pHrodo-TDP-43_agg_ + IgG2c ctrl vs. pHrodo-TDP-43_agg_ + 36C10 (G2c): *p* = 0.023; non-treated vs. pHrodo-TDP-43_agg_: *p* = 0.023; pHrodo-TDP-43_mono_ vs. pHrodo-TDP-43_agg_: *p* = 0.023; TDP-43_agg_ vs. pHrodo-TDP-43_agg_: *p* = 0.023; pHrodo-TDP-43_agg_ vs. pHrodo-TDP-43_agg_ (22 °C): *p* = 0.023

Therefore, we established a flow-cytometry based assay using pHrodo-conjugated recombinant TDP-43, which shows increased fluorescence upon uptake in the endo-lysosomal compartment. To avoid interference of antibodies with aggregate formation, we induced aggregate formation of pHrodo-conjugated TDP-43-MBP-His_6_ using TEV cleavage (for 30 min) and then incubated the samples with individual mAbs at equimolar ratios for additional 30 min before adding them to SH-SY5Y cells (Fig. 8A). Unlabeled TDP-43 and incubation at 22 °C to inhibit endocytosis served as negative controls. Importantly, we noticed significant uptake of preaggregated pHrodo-TDP-43 compared to the background defined by unlabeled TDP-43. Moreover, incubation at 22 °C reduced the signal nearly to the background level determined using unlabeled TDP-43 (Fig. 8B/C). To confirm the effects of mAbs on TDP-43 uptake we preincubated monomeric and aggregated TDP-43 with mAbs as described above. 31E9 and to a lesser extent 36C10 reduced the number of pHrodo-TDP-43-positive cells, while the moderate effect of 36C5 did not reach statistical significance. We did not observe substantial uptake of monomeric pHrodo-conjugated TDP-43-MBP-His_6_, suggesting aggregation promotes cellular uptake.

Taken together, high-affinity antibodies targeting the RRM2 or the C-terminal low complexity domain potently suppress TDP-43 phase separation, aggregation, and cellular aggregate uptake, all processes implicated in TDP-43 proteinopathies.

## Discussion

We used an active immunization approach in a fast-progressing murine model of ALS/FTD to target TDP-43 proteinopathy with antibodies in vivo. To this end, we systematically analyzed the immunogenicity, safety, and efficacy of predicted immunogenic regions covering all major domains of TDP-43. Only vaccinating with a C-terminal epitope containing the Ser409/410 phosphorylation sites significantly reduced serum NfL in vivo. By generating a panel of monoclonal antibodies, we identified several clones directed against the RRM2 and the glycine-rich domain that potently inhibited TDP-43 aggregation and phase separation in vitro, events tightly linked to neurodegeneration in ALS/FTD [3, 36].

### TDP-43 mouse model

The inducible rNLS8 model mimics gain- and loss-of-function components of TDP-43 proteinopathies, reflected by prominent cytoplasmic aggregation and accompanying nuclear clearance of TDP-43 [56]. Although aged rNLS8 mice become symptomatic at a similar time point after transgene induction as young rNLS8 mice, their disease progression is accelerated compared to young animals, resulting in a ~ 30% shorter lifespan [51]. In line with these findings, the lifetime of many proteins increases in the brains of old mice and aggregation-prone and low-complexity domain-harboring proteins, including TDP-43, are one of the most stabilized groups [23]. Based on our experience with anti-GA immunization [63], we had chosen a four-month immunization regimen to achieve high antibody titers, which required inducing the transgene in aged, fast-progressing rNLS8 mice. However, the anti-TDP-43 immune response already plateaued after two immunizations and future studies could be done in younger animals.

Moreover, immunofluorescence and transcriptomics revealed pronounced microgliosis by three weeks after transgene induction. In young animals, profound microgliosis has only been reported in the recovery stage, i.e., after switching off transgene induction [50], while transcriptomic profiling suggested microglial activation in older animals already after inducing transgene expression, as seen in our hands [19]. Thus, aging may influence the microglial response to TDP-43 aggregation, which should be taken into account for future mouse studies of ALS/FTD. Therefore, analyzing the effects of TDP-43 immunotherapy in younger animals using passive immunization might reveal effects of the therapy masked by the aggressive progression of disease in aged rNLS8 mice.

As in previous reports, we used the rNLS8 model in a mixed genetic background resulting from crossing F1 C57BL/6J × C3H/HeJ hybrid parents [56], which may explain the large variance observed in transcriptome analysis even in the control groups. While principal component analysis could clearly separate rNLS8 mice from monogenic control mice, the remaining variance could not be explained by the antigen groups or gender. This high variability may explain why we could not detect a single gene that was significantly changed upon immunization after FDR correction, although transcriptome-wide expression differences show a subtle but significant rescue of transgene-induced changes. Backcrossing the rNLS8 line to an inbred genetic background may reduce variability and thus increase statistical power in future therapeutic studies. Indeed, rNLS8 mice on a pure C57Bl/6JAusb background were used in a recent study and found to be phenotypically consistent with the originally described mixed background mice [56, 62].

### Safety and immunogenicity of TDP-43 peptide antigens

TDP-43 is a potentially risky drug target because its nuclear levels need to be maintained in a narrow range and it is required for regular cellular function in all cell types [6, 42]. In our immunization regimen, only the phospho-peptide (#15) induced disease-specific antibodies, while even the aggregated peptide antigens (peptides 7, 8, 10) induced antibodies detecting physiological TDP-43 in the nucleus. Moreover, five peptide antigens (5, 7, 8, 11, and 12) are not fully conserved between human and mouse TDP-43 and may have induced antibodies preferentially targeting human TDP-43 but sparing mouse TDP-43, as it has been observed for mAbs [24]. Thus, the residual expression of endogenous mouse TDP-43 together with the human transgene in rNLS8 mice may occlude potential loss-of-function toxicity induced by these antibodies in cells expressing only human TDP-43.

While most screened peptide antigens induced detectable antibody response in C57BL/6J mice, we noticed striking differences in the antibody titer unrelated to the peptide length. Thus, we used only the most immunogenic peptides from our pilot study for therapeutic studies in rNLS8 mice. To our surprise, we noticed unexpected toxicity for the highly immunogenic N-terminal TDP-43 peptides 1 and 3 in the mixed C57BL/6J × C3H/HeJ background in contrast to the pilot study in C57BL/6J mice. As most of the TDP1 + 3 animals unexpectedly died or had to be sacrificed shortly after the second or third immunization, we suspect that an anaphylactic reaction or local intraperitoneal toxicity occurred, rather than on-target toxicity due to gradually accumulating TDP-43 antibodies interfering with endogenous TDP-43 function. Interestingly, C3H/HeJ mice (but not the otherwise genetically similar C3H/HeOuJ strain) carry a loss-of-function mutation in *Tlr4*, the receptor for bacterial lipopolysaccharide. Loss of *Tlr4* has been discussed as a cause for stronger anaphylactic response to peanut antigen in C3H/HeJ mice compared to C3H/HeOuJ and Balb/c mice [8], but other genetic differences in the mixed C57BL/6J × C3H/HeJ background may also promote anaphylaxis towards peptides 1 + 3 in this model. Although we cannot fully explain the different safety profiles in these lines, our findings highlight the importance of considering genetic background-specific variations for preclinical trials in rodent models.

### Efficacy of TDP-43 active vaccination

To investigate the efficacy of anti-TDP-43 vaccination, we analyzed body weight, pTDP-43 levels, and serum NfL levels, a widely used clinical biomarker for neuroaxonal damage. While we did not observe beneficial effects on weight loss and pTDP-43 levels, immunization with the disease-associated C-terminal phospho-TDP-43 epitope significantly reduced NfL levels. These findings support the disease-promoting role of TDP-43 phosphorylation at serines 409/410, although the effects of C-terminal phosphorylation on TDP-43’s biophysical properties are controversially debated based on in vitro data [2, 16, 43]. We also investigated altered neuroinflammation upon vaccination, because microglia are emerging as key modulators in multiple neurodegenerative diseases, including TDP-43 proteinopathies [58], and active vaccination targeting poly-GA in our *C9orf72* ALS model affected microglia activation more than poly-GA levels [63]. While anti-TDP-43 immunized mice still showed pronounced microgliosis and astrogliosis as well as elevated chemokine levels, transcriptomics revealed a small overall reduction of the disease signature.

One postulated mode of action for antibody-based therapy is the prevention of cell-to-cell propagation of transmittable species of aggregation-prone proteins. Transmission has been extensively shown for proteins implicated in various neurodegenerative diseases, including Tau, α-synuclein, and for TDP-43 [9, 15, 39, 43]. Interestingly, two mAbs targeting the C-terminal domain efficiently prevented cellular uptake of preformed TDP-43 aggregates in neuron-like cells. A possible explanation for the modest effect of TDP-43 vaccination in rNLS8 mice may be the high and widespread deposition of TDP-43 aggregates in this model, which clearly exceeds the pathology in human ALS/FTD and cannot recapitulate focal onset and spreading of TDP-43 pathology across different brain regions over time [9, 48]. Since 95% of neurons in cortical layer 5 are found to be hTDP-43-positive already one week after transgene induction [56], it seems unlikely that spreading of TDP-43 species plays a dominant role in pathogenesis in the rNLS8 mouse model and therefore therapeutic antibodies cannot counteract this process. Very recently, systemic administration of a mAb targeting the C-terminal domain of TDP-43 was shown to reduce pTDP-43 levels and microgliosis in rNLS8 mice and neuron loss using the slower-progressing Camk2a-tTA model [1]. Previously, AAV-based expression or intrathecal injection of TDP-43 antibodies could ameliorate phenotypes in a BAC-transgenic TDP-43 mouse model but this model has a far slower progression with less abundant TDP-43 pathology than the rNLS8 mice [44, 45].

### mAbs can inhibit key disease features of TDP-43 in vitro

As passive antibody therapy may overcome weak immunogenicity of crucial epitopes and avoid acute toxicity, we generated a large panel of monoclonal TDP-43 antibodies targeting several of our selected epitopes. Four out of nine novel high-affinity antibodies targeting RRM2 (peptide 8, amino acids 198–211), the C-terminal glycine-rich region (peptide 12, aa. 342–379) or the linking region (peptide 10, aa. 258–296) strongly inhibited aggregation and altered phase separation of recombinant TDP-43 in different assays, while antibodies binding TDP-43 further N-terminal were less effective. These findings are consistent with the dominant role of the C-terminal glycine-rich domain in driving phase separation and aggregation of TDP-43 [7, 11, 13, 17]. A recent cryo-electron microscopy structure of TDP-43 aggregates extracted from ALS/FTD cortices revealed an ordered filament core comprised of aa. 282–360 [4], highlighting the importance of this glycine (G), glutamine (Q), and asparagine (N)-rich region for pathological TDP-43 aggregation. The significance of the Q/N-rich region (within the glycine-rich domain) for TDP-43 aggregation is further emphasized by the finding that tandem repeats of residues 331–369 trigger the formation of phosphorylated and ubiquitinated TDP-43 aggregates in cultured cells [10]. The overlap of our peptides 10 (aa. 258–296) and 12 (aa. 342–379) with these regions possibly explains the extraordinary aggregation-suppressing effect of the monoclonal antibodies raised against these regions.

Interestingly, antibodies targeting the glycine-rich region either resulted in the formation of much smaller and fewer TDP-43 condensates (31E9) or caused the formation of larger liquid-like droplets enriched in anti-TDP-43 antibodies (36C5 and 36C10), reminiscent of the phase separation behavior of a TDP-43 mutant containing multiple phospho-mimetic substitutions in the C-terminal domain [16]. Collectively, these findings suggest that disturbing the self-self-interaction of the C-terminal low complexity domain, either by hyperphosphorylation or high-affinity antibodies, suppresses both TDP-43 phase separation and aggregation, thereby supporting the fundamental role of TDP-43 C-terminal region for these processes.

Moreover, we show that aggregated TDP-43 is preferentially taken up by neuron-like cells compared to monomeric TDP-43, which can be blocked efficiently by two mAbs targeting the C-terminal domain (31E9 and 36C10), but not by 30D3 targeting the RRM2 domain. Interestingly, Afroz et al. show that a mAb targeting the C-terminal domain can inhibit seeding from patient material, promote phagocytic clearance by microglia and inhibit aggregate formation in vitro [1], but they have not analyzed effects on phase separation or neurofilament levels. Together with our findings, these data highlight the importance of the C-terminal region in the process of cell-to-cell transmission of aggregates.

## Conclusions

Taken together, we addressed efficacy and safety risks of TDP-43 immunotherapy for ALS/FTD by unbiased epitope screening in vitro and in vivo. We identified several highly immunogenic peptides in TDP-43 and raised a panel of novel mAbs potentially useful for therapy. Despite concerns about on-target toxicity of anti-TDP-43 antibodies due to the essential role of TDP-43 for cellular survival, we only detected unexpected safety risks of highly immunogenic N-terminal peptides, presumably due to anaphylaxis in one of four genetic backgrounds tested. In a fast-progressing TDP-43 mouse model, targeting the glycine-rich domain was most beneficial and partially rescued the disease signature detected by transcriptomics. While none of the antigens reduced the effect on neuropathology and the characteristic body weight loss, vaccination with a peptide including the disease-associated pS409/410 site significantly reduced NfL levels, the gold standard biomarker for ALS progression. Moreover, we identified mAbs targeting the glycine-rich domain that efficiently block disease-related phase separation and aggregation of TDP-43 in vitro, and suppress aggregate uptake in cells. Spreading and seeding of TDP-43 are crucial for the clinical progression of ALS after focal onset, but we assume it may be less relevant in the fast-progressing rNLS8 mice with widespread neuronal protein expression. Our finding that mAbs can alter the phase separation properties of a disease-linked protein, establishes a new mode of action for antibody therapy. Our study suggests that targeting the glycine-rich domain with antibodies ameliorates key pathomechanisms of TDP-43 proteinopathies.

## Supplementary Information

**Additional file 1: Figure S1**. TDP-43 epitopes and preimmune control serum. A: Overview of peptide antigens conjugated with ovalbumin through the N-terminal cysteine residue. Note that OVA conjugates of peptides 7, 8, 10, and 11 showed some aggregation in vitro. Peptides in bold were highly immunogenic in C57BL/6J wild-type mice and were thus used for the treatment study in rNLS8 mice. Novel monoclonal antibodies were raised in C57BL/6J mice, except of 26A2 and 26F12, which were generated from immunized Balb/c mice. B: Preimmune serum contains no detectable antibodies against human recombinant TDP-43 or pTDP-43 peptide as determined by ELISA. Background-corrected mean optical density at 450 nm was measured in duplicates per animal. All sera were diluted 1:100. **Figure S2**. Combined TDP-43 peptides are equally immunogenic and safe in three inbred mouse strains. Antibody response plateaus already after two immunizations. A: Timeline of immunization with pooled TDP-43 peptides, blood sampling, and body weight measurement of C57BL/6J, C3H/HeOuJ, and CD-1 mice. For each strain, six animals per treatment group were used. B: A dilution series of pooled sera was analyzed by ELISA against recombinant full-length TDP-43 or pTDP-43 peptide. Background-corrected mean OD450 was measured in duplicates. Triangular shapes indicate CD-1, circles represent C57BL/6J, and squares depict C3H/HeOuJ animals. Open shapes correspond to first blood sampling, while b2 is illustrated by filled shapes. C: Kaplan–Meier plot indicating survival probability of mice from different strains. No antigen combination caused statistically significant lethality in any of the strains investigated. D: Development of body weight over 14-week study period. Mean values are plotted group-wise. Only age, but not treatment or the interaction between treatment and age, had a statistically significant effect on the body weight as revealed by a two-way repeated measures ANOVA. C57BL/6J: p = 1.74*10^–24^; C3H/HeOuJ: p = 2.56*10^–33^; CD-1: p = 1.37*10^–29^. E: Dilution series of pooled rNLS8 sera from different time points were analyzed by ELISA against recombinant full-length TDP-43 or pTDP-43 peptide. Background-corrected mean OD450 was measured in duplicates. Note that the antibody response plateaued after two immunizations. Age of animals is annotated for each timepoint. **Figure S3**. Additional immunofluorescence stainings of pooled antisera confirm specificity towards nuclear and mislocalized TDP-43. Double immunofluorescence stainings of pooled antisera and with either phosphorylated TDP-43 or pan-TDP-43 were performed on frontal cortex sections of an FTLD case and a healthy control. A: Antisera from TDP1 + 3# and TDP10 + 12 animals reliably stained nuclear TDP-43 but no cytoplasmic inclusions in a control case negative for pTDP-43. B: Double immunofluorescence with a commercial TDP-43 antibody validates specificity of pooled antisera in patients. Antisera from TDP1 + 3^#^ and TDP10 + 12 mice detected both nuclear and cytoplasmic TDP-43, while TDP15 sera showed strong preference for cytoplasmic TDP-43 with little affinity to nuclear TDP-43. Representative images are shown. All scale bars = 10 µm. **Figure S4**. Additional comparisons of gene expression profiles and cytokine assays in rNLS8 mice. A: Expression patterns of genes characterizing early, late, and recovery stage in isolated microglia [19] in rNLS8 mice/ALS patients. Asterisks denote significant changes. LFC: log2-fold change. B: Concordant gene expression changes between rNLS8 mice and ALS patients. Only statistically significant genes with a LFC value ≥ 1 are shown. The 27 genes upregulated in both conditions cluster into different GO categories as depicted in Fig. 6C. C: Cytokine levels determined by multiplex assay from cortical tissue of vaccinated rNLS8 mice and controls as in Fig. 6E. Non-affected analytes are shown. From the panel INF-γ, IL-2, IL-4, IL-10, IL-12p70 could not be detected at all. IL-5 and IL-6: Control PBS n = 6, ctrl VAX n = 6, TDP-PBS n = 10, TDP5 + 7 n = 6, TDP8 + 9 n = 9, TDP10 + 12 n = 9, and TDP15 n = 7; IL-9, IL-15, IL-17A/F, IL-27p28/IL-30, IL-33, and MIP-2: Control PBS n = 10, ctrl VAX n = 10, TDP-PBS n = 10, TDP5 + 7 n = 13, TDP8 + 9 n = 12, TDP10 + 12 n = 12, and TDP15 n = 12. MIP-2 levels were only statistically significantly elevated in TDP-PBS compared to ctrl PBS mice (*p* = 0.004), but not ctrl VAX mice as indicated by pairwise t-test with Benjamini–Hochberg correction. D: RNAseq-based gene expression of all detected cytokines from the cytokine/chemokine panel in rNLS8 mice and ALS patients compared to controls as in Fig. 6A. Asterisks denote significant changes. **Figure S5**. Characterization of TDP-43 mAbs on droplet formation, affinity, and patient inclusions. A: Representative time-lapse images of Alexa 488-labeled TDP-43 condensates obtained by spinning disc confocal microscopy. TDP-43 treated with isotype control antibody does not fuse, while mAbs targeting the C-terminal glycine-rich region increase droplet mobility, leading to slow fusion events and enlarged TDP-43 droplets. Scale bar = 2.5 μm. B: Novel TDP-43 mAbs were analyzed by ELISA against recombinant TDP-43 in dilution series. Background-corrected OD450 are shown. C: mAbs which most potently inhibited TDP-43 aggregation and LLPS detected pTDP-43-positive inclusions in a FTLD patient and additionally stained nuclear TDP-43 by immunofluorescence.

**Additional file 2: Table S1**. Differential expression analysis of control PBS, TDP-PBS, TDP10+12, and TDP15 neocortex using DESeq2 as described in the methods. Samples are compared to either ctrl PBS or TDP-PBS. In column D “−1” and “+1” indicate significant genes and the direction of change.

**Additional file 3: Table S2**. Full Gene Ontology enrichment analysis for differentially expressed genes from TargetALS patients in spinal cord and genes expressed in rNLS8 mice, each both compared to non-diseased controls. Selected data are shown in Fig. 6C.

## Data Availability

All data supporting the conclusions of this article are included within the article and its supplementary materials. The raw data for the transcriptomics analysis are available under the GEO accession code GSE233669.

## Abbreviations

ALS: Amyotrophic lateral sclerosis
FTD: Frontotemporal dementia
FTLD-TDP: Frontotemporal lobar degeneration with TDP-43 inclusions
rNLS8 mice: ‘Regulatable’ or NEFH-tTA/tetO-hTDP-43ΔNLS bigenic mice
mAb: Monoclonal antibody
CNS: Central nervous system
Aβ: Amyloid beta
RRM1/2: RNA recognition motif 1/2
NLS: Nuclear localization sequence
NTD: N-terminal domain
LLPS: Liquid–liquid phase separation
scFv: Single-chain variable fragment
pTDP-43: TDP-43 phosphorylated at serines 409/410
i.p.: Intraperitoneally
s.c.: Subcutaneously
NfL: Neurofilament light chain
OVA: Ovalbumin
CSF: Cerebrospinal fluid
MBP: Maltose-binding protein
LPS: Lipopolysaccharide
RT: Room temperature
OD: Optical density
DOX: Doxycycline
ctrl: Control
MCtx: Motor cortex
SC: Spinal cord
