# Supplementary material for: Targeting the glycine-rich domain of TDP-43 with antibodies prevents its aggregation in vitro and reduces neurofilament levels in vivo
Source: Acta Neuropathol Commun. 2023 Jul 11;11:112. doi: 10.1186/s40478-023-01592-z (PMC10334564; doi:10.1186/s40478-023-01592-z)
Supplement: Supplementary file 1 — Additional file 1: Figure S1. TDP-43 epitopes and preimmune control serum. A: Overview of peptide antigens conjugated with ovalbumin through the N-terminal cysteine residue. Note that OVA conjugates of peptides 7, 8, 10, and 11 showed some aggregation in vitro. Peptides in bold were highly immunogenic in C57BL/6J wild-type mice and were thus used for the treatment study in rNLS8 mice. Novel monoclonal antibodies were raised in C57BL/6J mice, except of 26A2 and 26F12, which were generated from immunized Balb/c mice. B: Preimmune serum contains no detectable antibodies against human recombinant TDP-43 or pTDP-43 peptide as determined by ELISA. Background-corrected mean optical density at 450 nm was measured in duplicates per animal. All sera were diluted 1:100. Figure S2. Combined TDP-43 peptides are equally immunogenic and safe in three inbred mouse strains. Antibody response plateaus already after two immunizations. A: Timeline of immunization with pooled TDP-43 peptides, blood sampling, and body weight measurement of C57BL/6J, C3H/HeOuJ, and CD-1 mice. For each strain, six animals per treatment group were used. B: A dilution series of pooled sera was analyzed by ELISA against recombinant full-length TDP-43 or pTDP-43 peptide. Background-corrected mean OD450 was measured in duplicates. Triangular shapes indicate CD-1, circles represent C57BL/6J, and squares depict C3H/HeOuJ animals. Open shapes correspond to first blood sampling, while b2 is illustrated by filled shapes. C: Kaplan–Meier plot indicating survival probability of mice from different strains. No antigen combination caused statistically significant lethality in any of the strains investigated. D: Development of body weight over 14-week study period. Mean values are plotted group-wise. Only age, but not treatment or the interaction between treatment and age, had a statistically significant effect on the body weight as revealed by a two-way repeated measures ANOVA. C57BL/6J: p = 1.74*10–24; C3H/HeOuJ: p = 2 [file 40478_2023_1592_MOESM1_ESM.pdf]

A

#	peptide sequence	position in hTDP-43	domain	immunized in rNLS8 mice	antibody clone	isotype
**1**	**C-RVTEDENDEPIEIPSEDDG**	6-24	N	TDP1+3		
2	C-GLRYRNPVSQCMR	39-52	N			
**3**	**C-YPKDNKRKMDETDA**	77-90	N	TDP1+3	25C1	IgG2c
					27A3	IgG1
4	C-KRAVQKTSD	97-105	N			
**5**	**C-WKTTEQDLKEYFST**	113-126	RRM1	TDP5+7		
6	C-VKKDLKTGHSKGF	135-147	RRM1			
**7**	**C-TEYETQVKVMSQRHMIDGRWCDCKLPNSKQSQDEPLRSR**	153-191	RRM1	TDP5+7	26A2	IgG2a
					26F12	IgG1
**8**	**C-TEDMTEDELREFF**	198-211	RRM2	TDP8+9	30D3	IgG2c
**9**	**C-GEDLIIKGISVHISNA**	244-260	RRM2	TDP8+9		
**10**	**C-SNAEPKHNSNRQLERSGRFGGNPGGFGNQGGFGNSRGGG**	258-296	C	TDP10+12	31D9	IgG1
					31E9	IgG1
11	C-LGNNQGSNMGGGM	299-311	C			
**12**	**C-SQQNQSGPSGNNQNQGNMQREPNQAFGSGNNSYSGSNS**	342-379	C	TDP10+12	36C5	IgG2c
					36C10	IgG2c
13	C-SASNAGSGSGF	387-397	C			
14	C-SMDSKSSGWGM	404-414	C			
**15**	**C-SMDSKS(p)S(p)GWG**	404-413	C	TDP15		

B

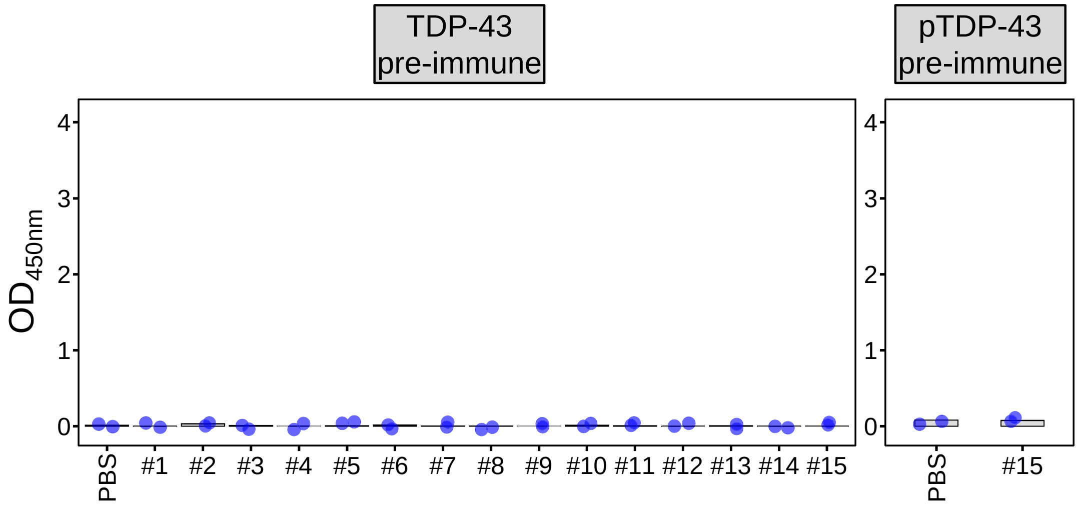

Figure S1

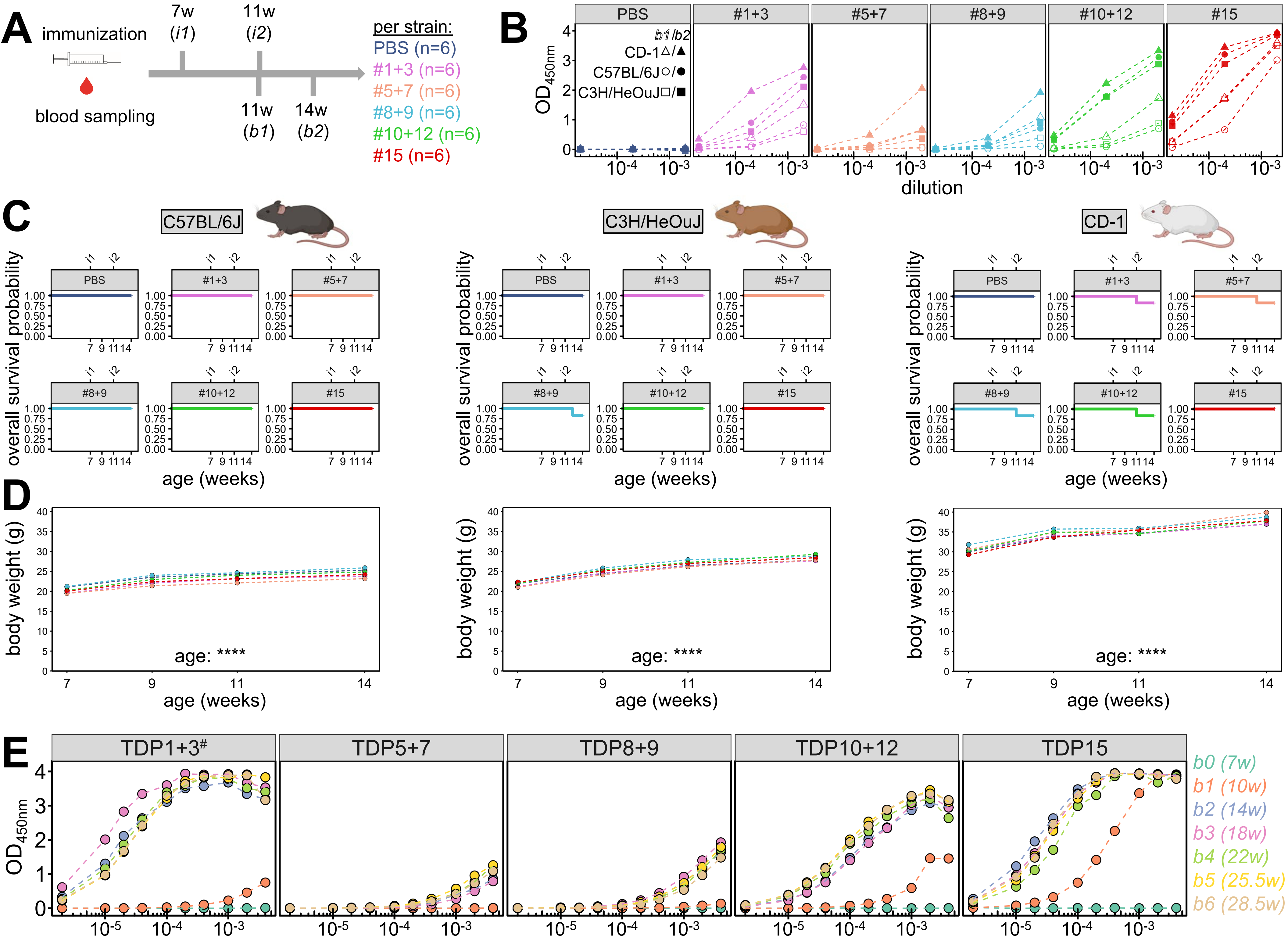
A
immunization
blood sampling
7w (i1)
11w (i2)
11w (b1)
14w (b2)
per strain:
PBS (n=6)
#1+3 (n=6)
#5+7 (n=6)
#8+9 (n=6)
#10+12 (n=6)
#15 (n=6)
B
PBS
#1+3
#5+7
#8+9
#10+12
#15
b1/b2
CD-1△/▲
C57BL/6J○/●
C3H/HeOuJ□/■
OD450nm
dilution
C
C57BL/6J
C3H/HeOuJ
CD-1
overall survival probability
age (weeks)
D
body weight (g)
age: ****
age (weeks)
E
TDP1+3#
TDP5+7
TDP8+9
TDP10+12
TDP15
OD450nm
dilution
b0 (7w)
b1 (10w)
b2 (14w)
b3 (18w)
b4 (22w)
b5 (25.5w)
b6 (28.5w)

Figure S2

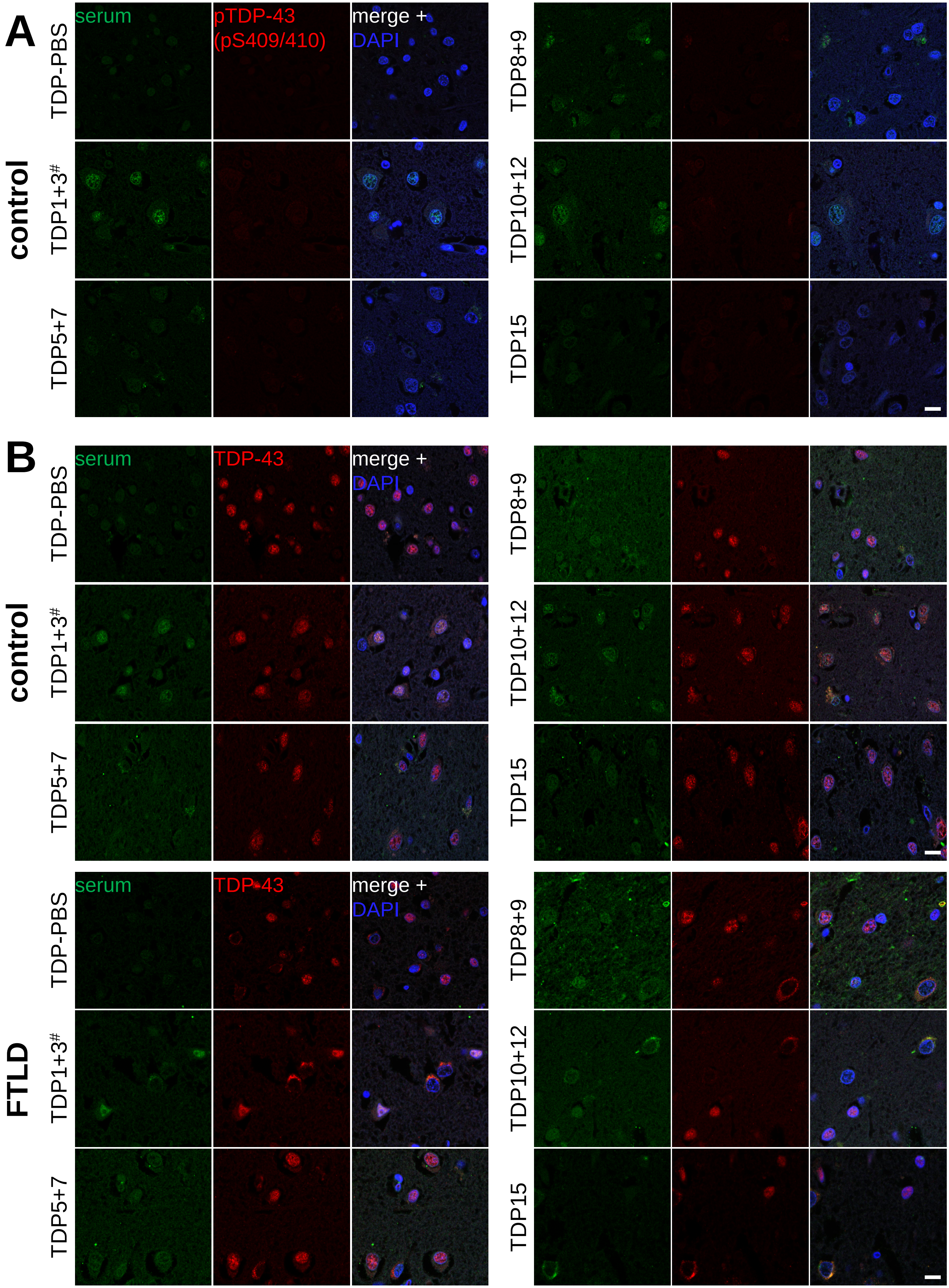
A
B
control
FTLD
serum
pTDP-43
(pS409/410)
TDP-43
merge +
DAPI
TDP-PBS
TDP1+3#
TDP5+7
TDP8+9
TDP10+12
TDP15

Figure S3

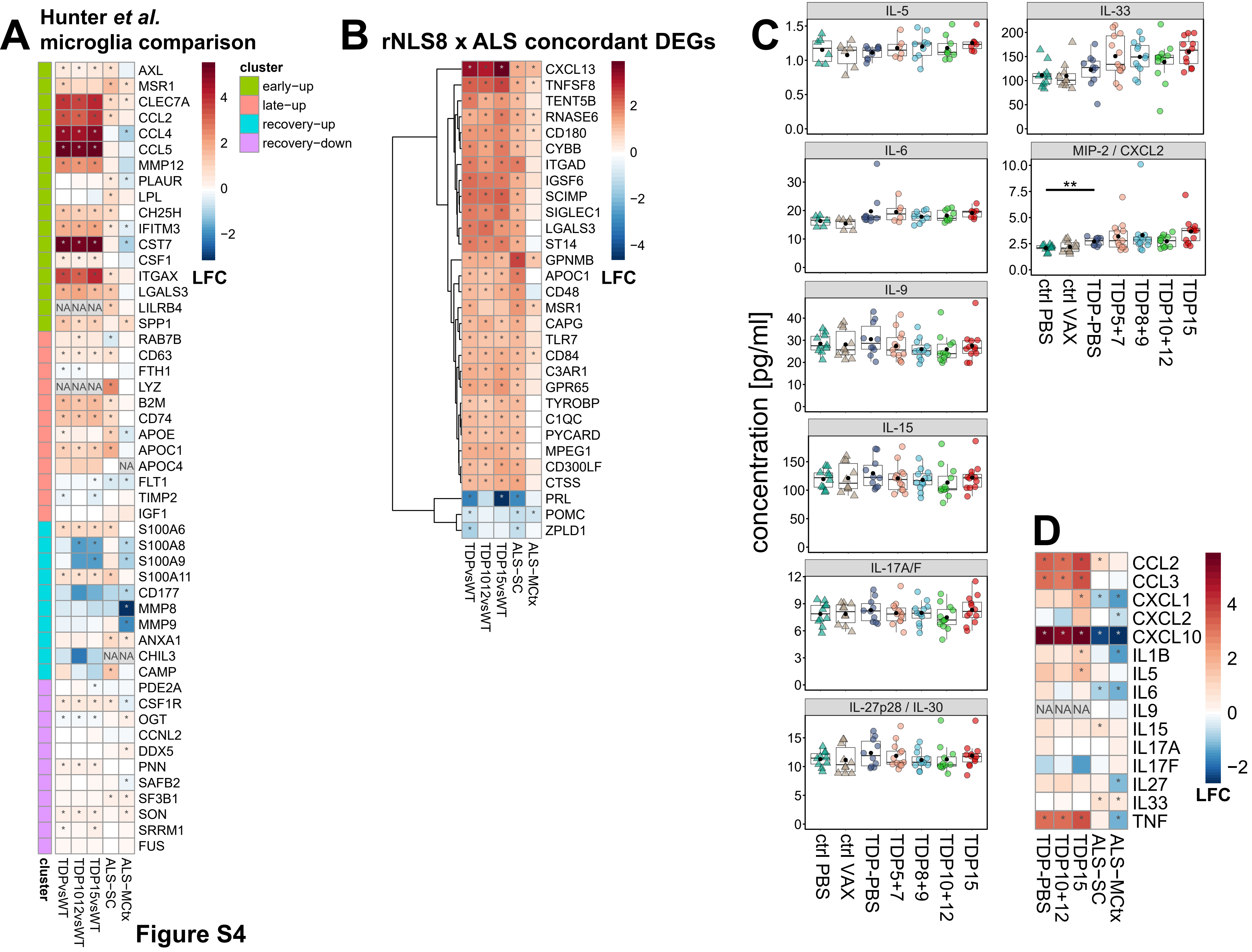

A
Hunter et al. microglia comparison
B
rNLS8 x ALS concordant DEGs
C
D
cluster
early−up
late−up
recovery−up
recovery−down
LFC
AXL
MSR1
CLEC7A
CCL2
CCL4
CCL5
MMP12
PLAUR
LPL
CH25H
IFITM3
CST7
CSF1
ITGAX
LGALS3
LILRB4
SPP1
RAB7B
CD63
FTH1
LYZ
B2M
CD74
APOE
APOC1
APOC4
FLT1
TIMP2
IGF1
S100A6
S100A8
S100A9
S100A11
CD177
MMP8
MMP9
ANXA1
CHIL3
CAMP
PDE2A
CSF1R
OGT
CCNL2
DDX5
PNN
SAFB2
SF3B1
SON
SRRM1
FUS
TDPvsWT
TDP1012vsWT
TDP15vsWT
ALS−SC
ALS−MCtx
CXCL13
TNFSF8
TENT5B
RNASE6
CD180
CYBB
ITGAD
IGSF6
SCIMP
SIGLEC1
LGALS3
ST14
GPNMB
APOC1
CD48
MSR1
CAPG
TLR7
CD84
C3AR1
GPR65
TYROBP
C1QC
PYCARD
MPEG1
CD300LF
CTSS
PRL
POMC
ZPLD1
IL-5
IL-33
IL-6
MIP-2 / CXCL2
IL-9
IL-15
IL-17A/F
IL-27p28 / IL-30
concentration [pg/ml]
ctrl PBS
ctrl VAX
TDP-PBS
TDP5+7
TDP8+9
TDP10+12
TDP15
CCL2
CCL3
CXCL1
CXCL2
CXCL10
IL1B
IL5
IL6
IL9
IL15
IL17A
IL17F
IL27
IL33
TNF
TDP-PBS
TDP10+12
TDP15
ALS−SC
ALS−MCtx

Figure S4

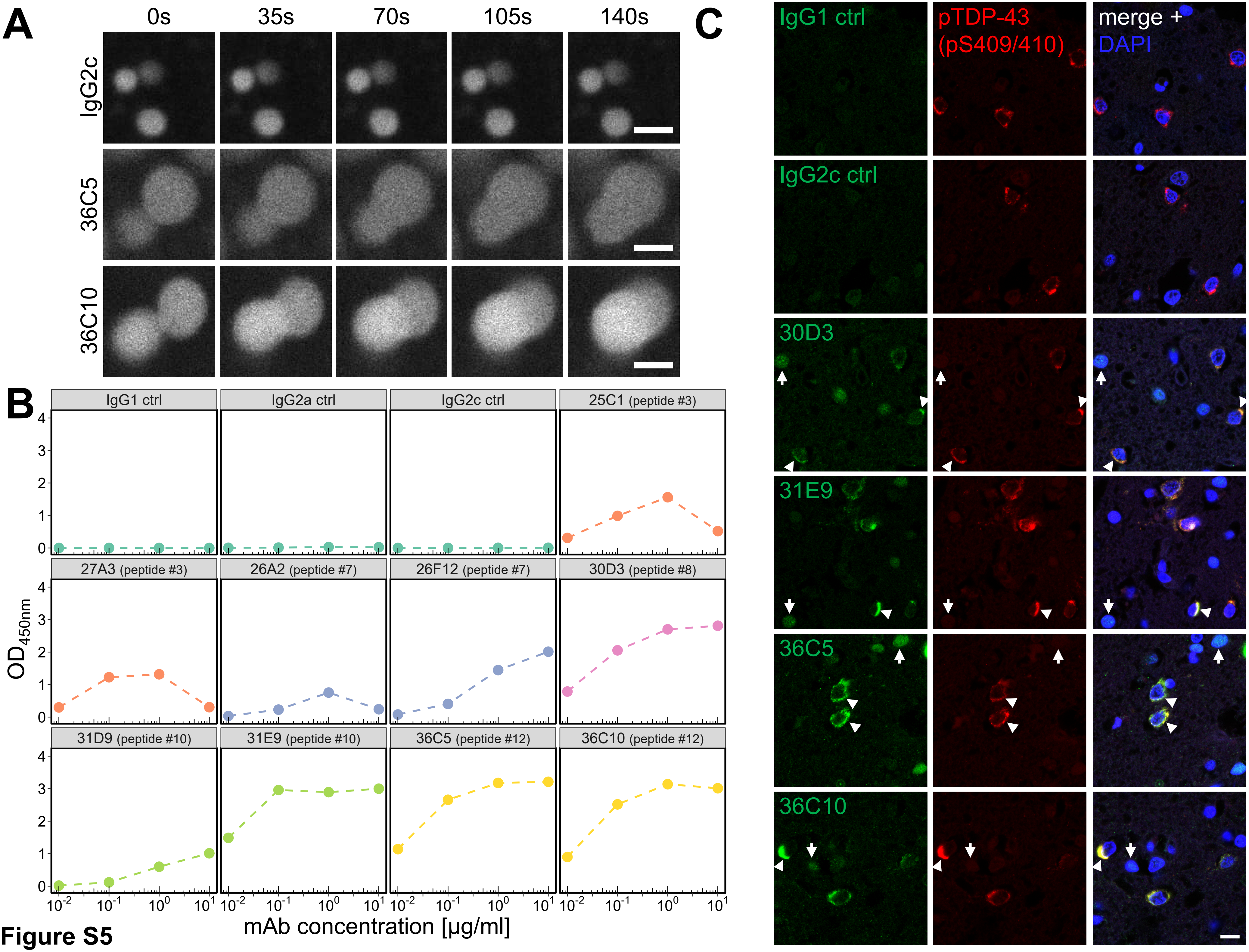
A
0s
35s
70s
105s
140s
IgG2c
36C5
36C10
B
IgG1 ctrl
IgG2a ctrl
IgG2c ctrl
25C1 (peptide #3)
27A3 (peptide #3)
26A2 (peptide #7)
26F12 (peptide #7)
30D3 (peptide #8)
31D9 (peptide #10)
31E9 (peptide #10)
36C5 (peptide #12)
36C10 (peptide #12)
OD450nm
mAb concentration [µg/ml]
C
IgG1 ctrl
pTDP-43 (pS409/410)
merge + DAPI
IgG2c ctrl
30D3
31E9
36C5
36C10

Figure S5
